# Wax-Functionalized Bacterial Cellulose Foils as Renewable Food Packaging with Enhanced Barrier and Antimicrobial Performance

**DOI:** 10.3390/polym18212705

**Published:** 2026-11-01

**Authors:** Elżbieta Redlarska, Klaudia Snopek, Martyna Mielniczuk, Karol Fijałkowski, Anna Żywicka

**Affiliations:** Department of Microbiology and Biotechnology, Faculty of Biotechnology and Animal Sciences, West Pomeranian University of Technology in Szczecin, Piastow 45, 70-311 Szczecin, Poland; elzbieta_lichwiarska@zut.edu.pl (E.R.); sk28850@zut.edu.pl (K.S.); mm56798@zut.edu.pl (M.M.);

**Keywords:** antimicrobial activity, bacterial cellulose, food packaging, food preservation, natural waxes, sustainable materials

## Abstract

This study investigated bacterial cellulose films (BCF) coated with twelve commercially available natural-origin wax and wax-like formulations as potential bio-based food-packaging materials. Bacterial cellulose (BC), produced by *Komagataeibacter xylinus*, was purified, homogenized, and dehydrated to obtain films suitable for subsequent wax coating. Among the preparation conditions investigated, a BC-to-water mass ratio of 1:2, a wet pulp mass of 45 g per dish, and drying at 60 °C for 24 h were selected. The resulting materials were evaluated for their water-related properties, surface wettability, antimicrobial activity, and antibiofilm performance. Wax coating reduced about threefold water uptake and liquid-water transfer compared with uncoated BCF and increased the mean water contact angle from 47.6° to 94.3°. The antimicrobial and antibiofilm effects varied depending on the wax formulation and the microorganism tested, reaching over 99% reduction in *C. albicans* and *S. aureus* for eight of the twelve wax formulations. Among the twelve formulations, the commercially available rapeseed–coconut wax blend (70:30; BCF-RCW) exhibited a favorable combination of water-related and antimicrobial properties and was selected for further surface micromorphology and mechanical characterization. In a preliminary food-storage experiment, all tested food samples wrapped in BCF-RCW exhibited significantly lower microbial counts than those wrapped in conventional polyethylene film or food wrapping paper during 21 days of refrigerated storage (over 99% reduction). These findings support the further development of wax-coated BC films as promising bio-based materials for functional food-packaging applications.

## 1. Introduction

The global production of plastics reached 413.8 million metric tons in 2023, with approximately 44% of this volume used by the packaging industry [1]. Petroleum-based synthetic polymers, such as polyethylene, polypropylene, polyethylene terephthalate, and polystyrene, remain the dominant packaging materials due to their durability, low cost, resistance to degradation, and excellent mechanical properties [2]. However, their limited biodegradability and poor recyclability pose serious environmental challenges, contributing significantly to long-term pollution [1].

Biodegradable polymers represent a promising alternative to conventional plastics, as they can decompose into water, carbon dioxide, and biomass under appropriate environmental conditions [3]. Both synthetic biopolymers, such as polylactic acid, polyhydroxyalkanoates, and bio-based polyesters, and natural biopolymers including starch, alginate, cellulose, and chitosan have demonstrated significant potential as sustainable substitutes for traditional, non-biodegradable packaging materials [4]. However, their broader commercial adoption remains limited due to inherent drawbacks, including inferior barrier properties, low thermal resistance, insufficient mechanical strength, and relatively high production costs. Overcoming these limitations is critical for positioning biodegradable polymers as viable mainstream packaging solutions [3].

Bacterial cellulose (BC) is a natural biopolymer produced by various environmental bacteria, including species of *Acetobacter*, *Pseudomonas*, *Komagataeibacter*, and *Agrobacterium*. Among these microorganisms, *Komagataeibacter xylinus* is one of the best-known and most widely used species for industrial BC production due to its high cellulose production yield [5]. BC, produced extracellularly by *K. xylinus*, has gained considerable attention as a sustainable material for food packaging due to its renewability, biocompatibility, and excellent mechanical properties. Its biodegradability under soil conditions has also been experimentally demonstrated, further supporting its potential as an environmentally sustainable packaging material [6]. However, its lack of intrinsic antimicrobial activity limits its direct application in active packaging systems. This limitation has prompted extensive research into BC functionalization for bioactive packaging applications. Thanks to its highly porous, interwoven nanofibrous architecture, BC provides an ideal scaffold for the incorporation or entrapment of antimicrobial agents [7]. Previous studies have demonstrated successful impregnation of BC with various antimicrobial substances, such as silver nanoparticles [8], carbon dots [9], and natural agents including chitosan and turmeric [10] or fermented soymilk [11].

The increasing global concern over microbial resistance to conventional chemical agents [12], combined with the demand for environmentally sustainable food packaging, underscores the need for greener strategies for BC surface modification. In addition to packaging intended for direct food contact, antimicrobial agents must remain immobilized within the material to prevent migration into food, thereby avoiding negative impacts on sensory quality and consumer safety [13].

The natural waxes and related lipid-based formulations represent a promising approach to BC surface modification, offering opportunities to improve moisture resistance and introduce additional functional properties. Previous studies have demonstrated the biodegradability of selected natural wax-based coatings under defined environmental conditions [14]. Furthermore, Chen et al. [15] showed that BC-based packaging films modified by oil infusion retained their biodegradability under the investigated conditions. Together with the established biodegradability of BC, these findings provide a scientific basis for exploring natural-origin hydrophobic coatings in the development of more sustainable food-packaging materials. However, the biodegradation behavior of a given BC–wax composite depends on its specific composition and structure and requires experimental verification.

Although natural waxes have been widely investigated as hydrophobic modifiers of biopolymer-based packaging materials, studies specifically addressing their combination with BC remain relatively limited. Indriyati and Indrarti [16] developed BC-based films incorporating beeswax, carboxymethyl cellulose, and a surfactant, focusing primarily on their physicochemical and mechanical properties. Subsequently, Indriyati et al. [17] demonstrated that beeswax incorporation improved the hydrophobicity and elasticity of BC-based films, although tensile strength decreased. Using a different approach, Frota et al. [18] developed superhydrophobic beeswax-based coatings reinforced with SiO_2_-functionalized BC nanofibrils, demonstrating pronounced water repellency relevant to food-packaging applications. More recently, Mariani et al. [19] investigated BC films coated with carnauba wax, showing that multilayer coating and hot pressing could improve surface hydrophobicity and water resistance. While emulsion-based approaches, including conventional and Pickering emulsions, offer alternative strategies for incorporating hydrophobic components into cellulose-based materials, direct surface coating allows molten waxes to be applied to preformed BC films without the need for emulsion preparation or additional emulsifying agents.

Collectively, these studies demonstrate the potential of combining BC with natural waxes to improve the functional properties of packaging materials. However, previous investigations have predominantly focused on physicochemical, structural, mechanical, and water-related properties, while the antimicrobial and antibiofilm performance of BC–wax composites remain comparatively underexplored. Moreover, systematic comparisons of multiple wax formulations, particularly in combination with evaluations of their effectiveness under food-storage conditions, remain scarce.

To address these research gaps, the present study investigates twelve commercially available natural waxes and wax blends as surface coatings for bacterial cellulose films (BCF), with particular emphasis on their water-related, antimicrobial, and antibiofilm properties. A dedicated homogenization and dehydration process was developed to convert BC pellicles into relatively uniform films suitable for subsequent wax coating. The resulting materials were characterized in terms of their physicochemical and biological properties, while the selected formulation was subjected to additional structural and mechanical analyses. Finally, a preliminary proof-of-concept experiment was conducted to evaluate its potential for preserving selected food products under refrigerated storage conditions.

## 2. Materials and Methods

### 2.1. Production of Bacterial Cellulose

BC was produced using the reference strain *Komagataeibacter xylinus* (American Type Culture Collection, ATCC 53524). The liquid Hestrin–Schramm (H–S) culture medium contained 20 g/L D-glucose, 5 g/L peptone, 5 g/L yeast extract, 1.15 g/L citric acid, and 2.7 g/L disodium hydrogen phosphate. The medium was supplemented with 1% (*v*/*v*) ethanol, and no agar was added. The initial pH was adjusted to 6.0 before sterilization.

A volume of 100 mL of H–S medium was dispensed into each sterile Petri dish (15 cm in diameter and 2 cm in height; Becton Dickinson and Company, Franklin Lakes, NJ, USA). Each dish was inoculated with 2 mL of a bacterial suspension containing 3 × 10^5^ colony-forming units per milliliter (CFU/mL), resulting in a final inoculum concentration of approximately 6 × 10^3^ CFU/mL.

BC biosynthesis was carried out under static conditions at 28 °C for 7 days, with the Petri dishes covered throughout the incubation period. Following cultivation, the BC pellicles were purified to remove bacterial cells and residual culture medium components. Purification was performed by immersing the pellicles in 0.1 M sodium hydroxide (NaOH) at 80 °C for 90 min, followed by immersion in deionized water. The water was replaced daily for several consecutive days until the pH of the water surrounding the BC pellicles reached neutrality [20]. The purified BC was subsequently sterilized and stored at 4 °C until further use.

### 2.2. Optimization of BC Films Production Parameters

Purified BC pellicles were cut into approximately 5 × 5 cm pieces using sterile scissors and homogenized with distilled water using a laboratory blender (Perfectmix+ BL811D38, Tefal, Rumilly, France; rated power: 1200 W; manufacturer-specified maximum rotational speed: 28,000 rpm). Three BC-to-water mass ratios (1:1, 1:2, and 2:1) were investigated. These ratios were calculated on a wet-mass basis using the mass of the purified, hydrated BC pellicles before homogenization.

Homogenization was performed at the maximum speed setting in three 15 s cycles, separated by approximately 2 min intervals to limit excessive heating. If visible fragments of the original BC pellicles remained, the homogenization procedure was repeated after a 5 min resting period. This procedure was continued until a visually homogeneous pulp without discernible membrane fragments was obtained.

The resulting BC pulp was weighed and transferred into round Petri dishes (60 mm in diameter) at three different masses: 30, 45, or 60 g per dish. These amounts were selected to evaluate the influence of the initial wet pulp loading on film formation and macroscopic uniformity. Since both the BC-to-water ratio and the amount of deposited pulp were defined on a wet-mass basis, these parameters describe the initial processing conditions rather than a standardized dry-cellulose loading.

The pulp was subsequently dried at 60, 70, or 80 °C for 24 h to obtain bacterial cellulose films (BCF). These temperatures were selected to investigate the influence of thermal dehydration conditions on film formation, surface uniformity, and flexibility, and to identify suitable processing conditions for subsequent wax coating. The selection was based on a comparative assessment of the materials obtained within the investigated temperature range. The effects of the BC-to-water mass ratio, the amount of pulp deposited per dish, and the drying temperature were evaluated based on the macroscopic appearance. For each experimental condition, three independently prepared films were evaluated.

For comparison, intact purified BC pellicles were also subjected to two alternative dehydration procedures. In the first procedure, the pellicles were dried directly at the temperatures specified above. In the second procedure, the pellicles were frozen at −20 °C for 24 h and subsequently freeze-dried at −60 °C under a pressure of 0.1 mbar (Alpha 1–2 LDplus, Martin Christ Gefriertrocknungsanlagen GmbH, Osterode am Harz, Germany). These samples were used to qualitatively compare the macroscopic appearance and handling characteristics of films prepared using different methods.

### 2.3. Preparation of Wax-Coated Bacterial Cellulose Films

The prepared BCF were coated with twelve commercially available wax and wax-like formulations of plant or animal origin, resulting in wax-coated bacterial cellulose films (BCF-W). The materials were selected based on their commercial availability, natural origin, and differences in physical characteristics, to enable a comparative assessment of their suitability for BC surface modification.

The following commercial formulations were investigated: soy wax (SW), olive wax (OW), laurel wax (LW), candelilla wax (CaW), coconut wax (CW), carnauba wax (CrW), rapeseed wax (RW), jojoba wax (JW), rapeseed–coconut wax blend (RCW), sunflower–olive wax blend (SOW), bleached beeswax (BBW), and pure beeswax (PBW). All materials were purchased from Ecoflores (Nowy Targ, Poland).

The commercial designation “wax” is used according to the supplier’s product nomenclature. Depending on the formulation, these materials may contain different lipid constituents, including wax esters, glycerides, fatty acids, and other components, and should not be considered chemically equivalent to the corresponding unmodified vegetable oils. The investigated products were used as supplied, without additional chemical processing or modification of their composition. According to the supplier’s specifications, RCW contained 70% rapeseed wax and 30% coconut wax, whereas SOW contained 50% sunflower wax and 50% olive wax. Both blends were purchased as ready-to-use formulations. No additional component ratios were prepared or evaluated, and optimization of the blend composition was not within the scope of this study. The available physicochemical characteristics of the formulations, including physical form, density, and melting temperature, are summarized in Table 1. Additional supplier-reported information on chemical composition and physicochemical parameters is provided in Supplementary Table S1. These specifications were not independently verified through chemical analysis in the present study.

**Figure 1:**
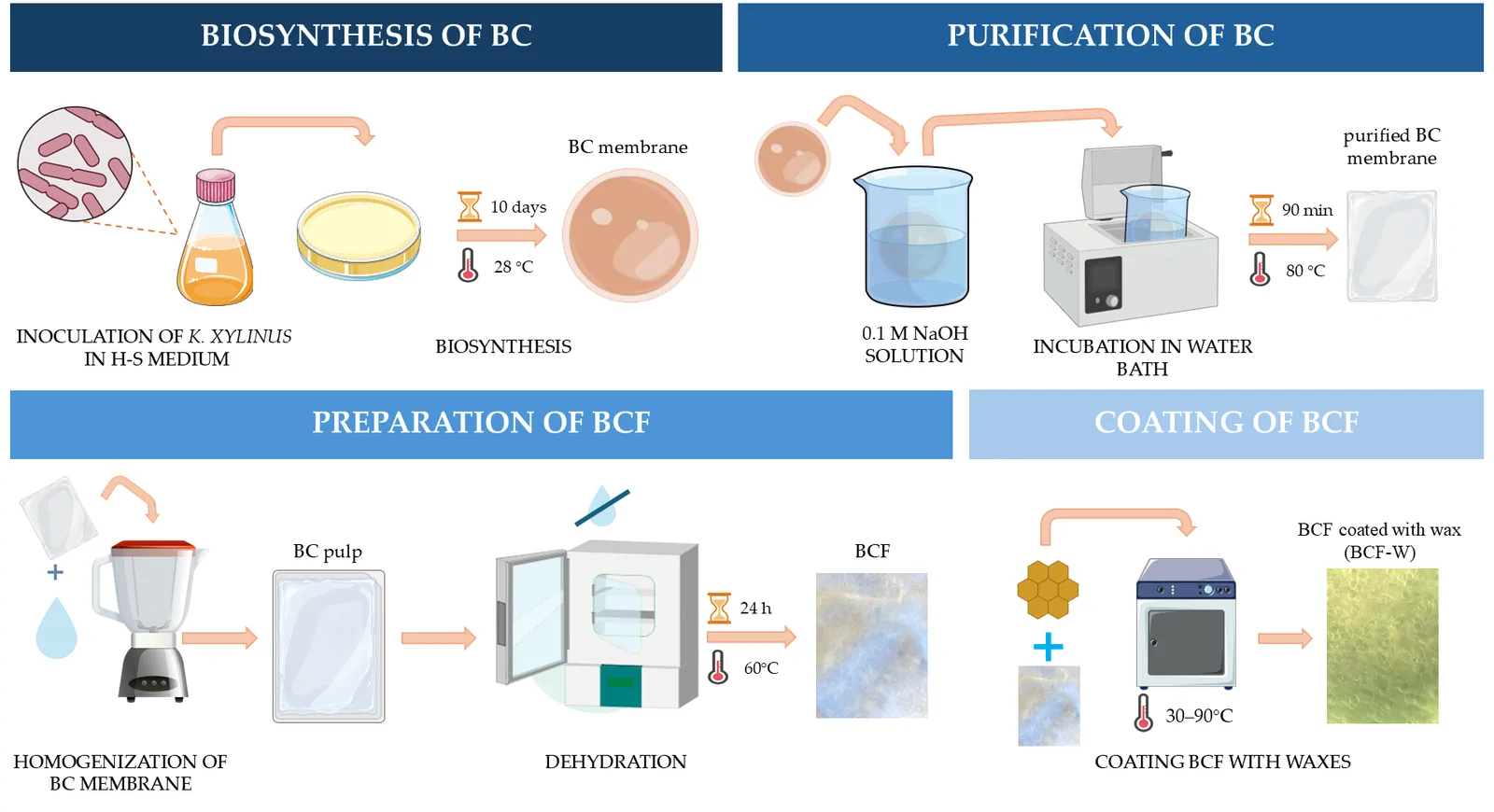
Schematic presentation of the preparation of bacterial cellulose films (BCF) and their coating with various waxes; bacterial cellulose (BC). Selected icons adapted from Servier Medical Art (CC BY 3.0 and CC BY 4.0) and NIAID Visual & Medical Arts (NIAID NIH BioArt Source, 2026); all other elements were created by the authors.

For the coating procedure, BCF discs (60 mm in diameter) were weighed using a laboratory balance (WTB 2000, Radwag, Radom, Poland). Portions of each wax formulation were distributed directly onto one surface of the BCF discs, without preliminary application using a brush. For each wax formulation, the amount required to achieve visually continuous coverage of both BCF surfaces was determined gravimetrically and expressed as wax loading per unit area (mg/cm^2^). Measurements were performed on three independently prepared wax-coated films per formulation, and the mean wax loading was calculated from these three independent determinations. The amount of wax varied depending on the formulation, ranging from 4.10 to 6.22 mg/cm^2^ (mean: 5.50 mg/cm^2^). The individual wax loading values are presented in Table 1. The wax was subsequently melted and spread across the film surface using a heated iron (Ultragliss 4870, Tefal, Rumilly, France). The heating conditions were adjusted according to the melting behavior of each wax formulation to facilitate spreading and obtain visually continuous surface coverage. The melting temperature ranges of the individual formulations are provided in Table 1. The same procedure was then repeated on the opposite side of each film to obtain double-sided wax coatings. A schematic illustration of the BCF preparation and wax-coating procedure is shown in Figure 1.

### 2.4. Macromorphological Characterization of BCF and BCF-W

The macromorphological characteristics of the BCF and BCF-W surfaces were examined using a stereoscopic microscope (Leica S9i, Leica Microsystems, Wetzlar, Germany) to assess surface appearance, color distribution, and visible defects.

Additionally, the conformability of BCF-W was assessed qualitatively by manually applying the films to the curved surfaces of laboratory tubes. Before application, the films were gently warmed using the heat of the hands to facilitate handling. The films were then manually fitted around the tubes, and their ability to conform to the curved surface was assessed based on visual observations of surface contact, visible cracking, and detachment. This assessment was qualitative and was intended to compare the handling and surface-conforming behavior of the different wax-coated films.

### 2.5. Water-Related Properties of BCF and BCF-W

#### 2.5.1. Swelling Ratio (*SR*%)

The swelling ratio (*SR*%) was determined using samples cut into 1 cm^2^ squares and weighed on an analytical balance (WTB 2000, Radwag, Radom, Poland). For each formulation, measurements were performed on three independently prepared film specimens (*n* = 3). Each sample was immersed in distilled water and removed at one-minute intervals, blotted with filter paper, and reweighed. The *SR* was calculated according to Equation (1):

(1) $$\mathrm{SR}\left[\%\right]=\left(\frac{m_{w}-m_{d}}{m_{d}}\right)\cdot 100$$

where *m_w_* is the wet mass of the BCF/BCF-W sample, and *m_d_* is the dry mass of the BCF/BCF-W sample.

#### 2.5.2. Liquid-Water Transfer (*WT*)

Liquid-water transfer (*WT*) was evaluated using a comparative gravimetric assay based on the absorbent-paper mass-gain principle of AATCC TM35 (Water Resistance: Rain). The procedure was modified to assess the response of small BCF specimens to direct liquid-water exposure rather than the controlled water spray specified in the standard method.

A piece of absorbent paper was weighed using a laboratory balance (WTB 2000, Radwag, Radom, Poland) and placed in a Petri dish beneath each BCF or BCF-W specimen (1 cm^2^). Subsequently, 1 mL of distilled water was applied to the upper surface of the specimen. The mass of the absorbent paper was determined at 1 min intervals over a 5 min period to quantify the amount of water collected beneath the film.

Liquid-water transfer was calculated according to Equation (2):

(2) *WT* (mg) = *m_t_* − *m*_0_

where *m_t_* is the mass of the absorbent paper at the specified measurement time and *m*_0_ is its initial dry mass.

During the measurements, the specimens were visually inspected for water leakage around their edges. Tests in which visible peripheral leakage occurred were excluded from the analysis. Three replicate measurements were performed for each material.

#### 2.5.3. Contact Angle

Surface wettability was evaluated by measuring the apparent static water contact angle using a DSA10 goniometer (Krüss GmbH, Hamburg, Germany) equipped with an automated drop-shape analysis system. Measurements were performed at room temperature (23.0–24.5 °C) using distilled water.

Water droplets with a volume of 2 µL were deposited onto the film surfaces using a microsyringe at a deposition rate of 110 µL/min. Droplet profiles were recorded using the instrument’s camera system, and contact angles were determined by numerical fitting of the droplet contours. The contact angle was measured 10 s after droplet deposition.

For each formulation, measurements were performed on three independently prepared films (*n* = 3), with five individual water droplets deposited at different locations on each film. The five contact angle measurements were treated as technical replicates and averaged to obtain a mean value for each film. The estimated instrumental measurement uncertainty was ±0.2°.

### 2.6. Antimicrobial and Anti-Biofilm Properties of BCF and BCF-W

#### 2.6.1. Antimicrobial Properties

The antimicrobial activity of the investigated materials was evaluated using a quantitative culture-based assay adapted for bacterial cellulose films and their wax-coated counterparts. The assay was performed against *Staphylococcus aureus* subsp. *aureus* (ATCC 6538, Gram-positive), *Escherichia coli* (ATCC 8739, Gram-negative), and the yeast *Candida albicans* (ATCC 10231). These microorganisms were selected as representatives of different microbial groups characterized by distinct cellular structures and biological properties.

Briefly, 0.4 g of each material was placed in a sterile Petri dish, and 200 µL of a microbial suspension containing 3 × 10^5^ CFU/mL was applied to the film surface. Bacterial suspensions were prepared in Brain Heart Infusion (BHI; Graso, Starogard Gdański, Poland) broth, whereas Sabouraud (SAB; Graso, Starogard Gdański, Poland) medium was used for *C. albicans*. The inoculated samples were incubated at 37 °C for 24 h. Uncoated BCF served as the reference control for evaluating the effects of wax modification, and the initial viable microbial concentration was verified by quantitative plating of the inoculum suspensions.

Following incubation, each sample was transferred to a 50 mL polypropylene conical centrifuge tube (Becton Dickinson and Company, Franklin Lakes, NJ, USA) containing 20 mL of phosphate-buffered saline (PBS; Sigma-Aldrich, Taufkirchen, Germany). The tubes were vortexed for 60 s to detach microorganisms from the film surfaces.

Microbial recovery was additionally assessed using an agar contact-imprint procedure. Immediately after vortexing, the tested films were brought into contact with the appropriate agar medium to assess the presence of residual culturable microorganisms on their surfaces. No colony growth was observed following incubation of the contact-imprint plates, supporting the effectiveness of the recovery procedure under the investigated conditions.

The integrity of the wax coatings was also assessed visually following vortexing, and no visible coating damage or detachment was observed. The recovery suspensions were subsequently subjected to serial tenfold dilutions and quantitatively plated onto BHI agar for the bacterial strains or SAB agar for *C. albicans*. Following incubation at 37 °C for 24 h, colonies were counted to determine the number of recoverable viable microorganisms.

Serial dilution was performed before plating to reduce the potential carryover of wax-derived substances into the enumeration step. However, the release of soluble wax constituents into the recovery medium and the possible effects of residual inhibitory activity on colony formation were not independently evaluated.

Antimicrobial effects were assessed by comparing the recoverable viable microbial counts obtained for wax-coated BCF with those obtained for uncoated BCF under the same experimental conditions. The antimicrobial assay was performed in three independent experiments for each material–microorganism combination, with duplicate plating of each recovered microbial suspension.

#### 2.6.2. Anti-Biofilm Properties

The anti-biofilm activity of the samples was assessed against the same microorganisms described in Section 2.6.1. The samples (0.1 g; WTB 2000, Radwag, Radom, Poland) were placed individually in 24-well plates (Nest Scientific Biotechnology, Woodbridge, NJ, USA). Each well was filled with 2 mL of a microbial suspension (3 × 10^5^ CFU/mL) prepared in BHI or, for *C. albicans*, SAB medium. Samples were incubated for 24 h at 37 °C. Following incubation, the medium was removed, and the samples were rinsed three times with deionized water to remove non-adherent cells. The remaining biofilm was stained with 0.4% (*w*/*v*) crystal violet solution (Sigma-Aldrich, St. Louis, MO, USA) for 10 min. Excess stain was removed by rinsing with deionized water. Following the final rinse, the samples were dried at 37 °C for approximately 30 min to minimize residual water before the addition of 30% (*v*/*v*) acetic acid (Chempur, Tarnowskie Góry, Poland) to solubilize the bound dye. This drying step was intended to prevent dilution of the extraction solution, which could otherwise introduce variability in absorbance measurements. The absorbance of the solubilized crystal violet was measured at 580 nm using a microplate reader (Infinite 200 PRO NanoQuant, Tecan, Männedorf, Switzerland). The anti-biofilm assay was performed in three independent experimental runs (*n* = 3), using a separate film specimen for each material–microorganism combination in each run. Absorbance was measured in triplicate for each specimen, and the mean absorbance value was calculated from these technical replicates.

### 2.7. Surface Micromorphology and Mechanical Characterization of BCF and BCF-RCW

Uncoated BCF and BCF coated with the rapeseed–coconut wax blend (BCF-RCW) were selected for further surface micromorphology and mechanical characterization. This formulation was selected because of its favorable combination of water-related and antimicrobial properties and its subsequent use in the preliminary proof-of-concept food-storage experiment.

#### 2.7.1. Micromorphological Assessment

The surface micromorphology of BCF and BCF-RCW was further examined using scanning electron microscopy (SEM; Apreo 2 S LoVac, Thermo Fisher Scientific, Waltham, MA, USA). Prior to examination, the samples were sputter-coated with a thin layer of gold to improve surface conductivity and reduce charging effects during imaging. Micrographs were acquired using an Everhart–Thornley detector (ETD) operating in secondary electron (SE) imaging mode, at accelerating voltages of 20–30 kV and a working distance of approximately 12 mm.

#### 2.7.2. Mechanical Characterization

The tensile properties of BCF and BCF-RCW were evaluated according to PN-EN ISO 527-3:2019-01 [21] guidelines using an Instron testing system (Norwood, MA, USA). Type 2 specimens were prepared as strips with nominal dimensions of 25 × 100 mm. No additional pressing or thickness-adjustment treatment was applied to the specimens before mechanical testing. The distance between the grips was set to 30 mm, and a preload of 0.5 N was applied. Tests were performed at a crosshead speed of 1 mm/min under ambient conditions of 22 °C and 50% relative humidity. Pneumatic grips were used.

The width and thickness of each specimen were recorded individually and used to determine its initial cross-sectional area for the calculation of engineering stress. Tensile strength, stress at break, strain at failure, and elastic moduli were obtained from the recorded force-displacement and stress–strain data. Maximum load was also recorded. Four specimens were tested for each material.

### 2.8. Preliminary Proof-of-Concept Food-Storage Experiment

A preliminary proof-of-concept experiment was conducted to evaluate the potential application of BCF-W as food-packaging materials under controlled refrigerated storage conditions. BCF-RCW was selected based on its favorable combination of water-related and antimicrobial properties among the BCF-W formulations investigated in this study. The performance of BCF-RCW was compared with that of conventional polyethylene film (PF) and food wrapping paper (FWP).

Individual 1 g portions of bread, apple, cheese, and ham were weighed using a laboratory balance (WTB 2000, Radwag, Radom, Poland) and separately wrapped in one of the three packaging materials. For each food product, packaging material, and sampling time, three independently packaged food portions were prepared and analyzed (*n* = 3). The packaged samples were stored at 4 ± 1 °C for 7, 14, or 21 days.

At each sampling time, individual food samples were transferred into sterile tubes containing 10 mL of phosphate-buffered saline (PBS) and vortexed for 3 min to recover microorganisms from the food material. The resulting suspensions were subjected to serial tenfold dilutions and quantitatively plated onto Brain Heart Infusion (BHI) agar. Following incubation at 37 °C for 24 h, the resulting colonies were counted. Microbial counts were calculated by accounting for the dilution factor, plated sample volume, and initial food sample mass, and expressed as colony-forming units per gram of food (CFU/g).

The assay was intended to provide a comparative assessment of culturable microbial counts recovered from food samples stored in contact with different packaging materials. The experiment was conducted as a preliminary screening using small food portions rather than as a simulation of commercial food-packaging conditions.

### 2.9. Statistical Analysis

When comparing multiple groups, data are expressed as mean ± standard error of the mean (SEM) calculated from the available experimental replicates. The number of independent experimental replicates and technical measurements depended on the assay and is specified in the corresponding methodological descriptions. Statistical differences between groups were evaluated using one-way analysis of variance (ANOVA) followed by Tukey’s post hoc test. For comparisons between two groups, data are expressed as mean ± standard deviation (SD) and Student’s *t*-test was used when the assumption of equal variances was met, whereas Welch’s *t*-test was applied when Levene’s test indicated unequal variances (*p* < 0.05). Differences were considered statistically significant at *p* < 0.05, while *p*-values between 0.05 and 0.10 were interpreted as indicating a statistical tendency. All statistical analyses were performed using GraphPad Prism version 9.0 (GraphPad Software Inc., San Diego, CA, USA).

## 3. Results and Discussion

### 3.1. Optimization of BCF Production Parameters

**Figure 2:**
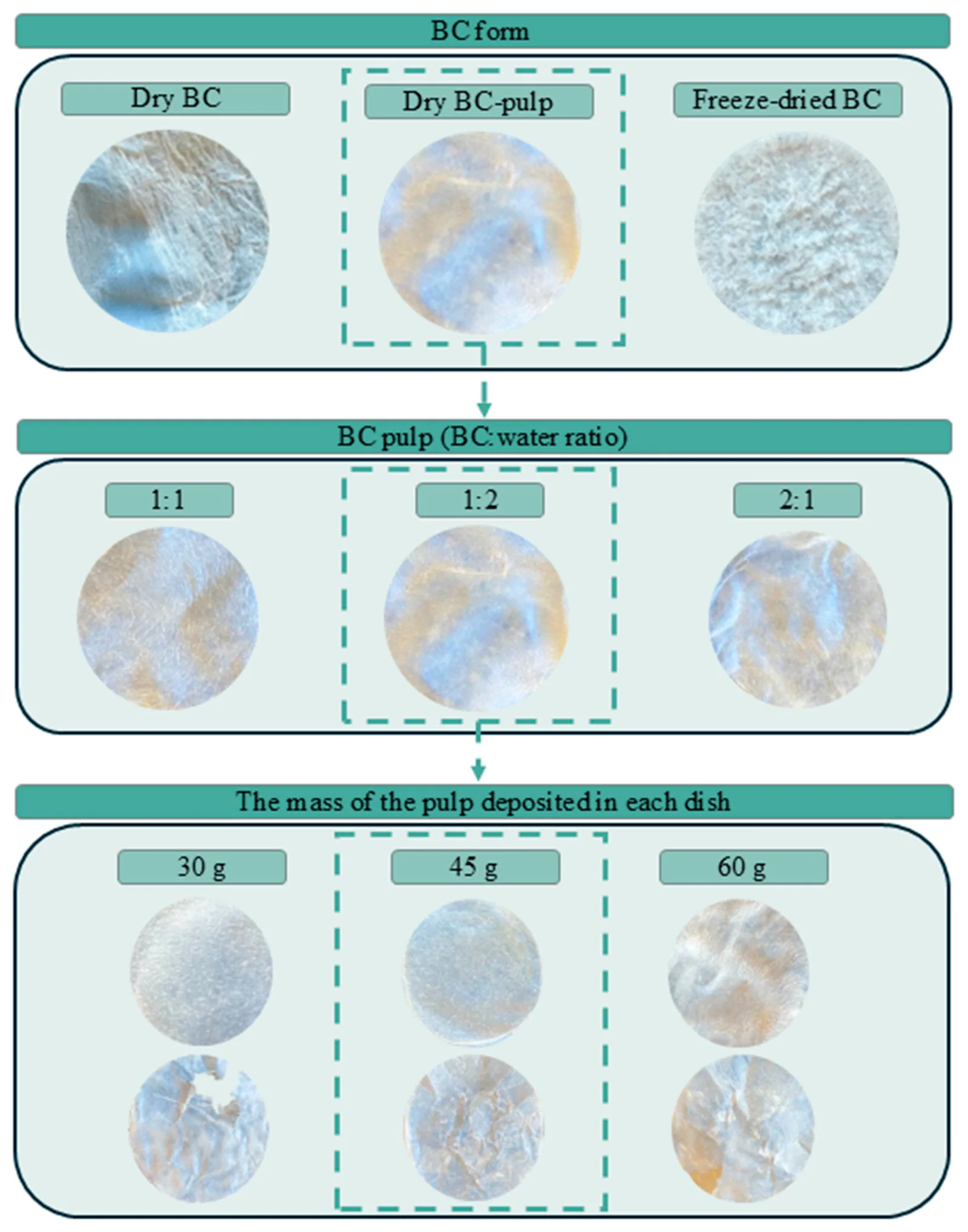
Macromorphological differences in bacterial cellulose films (BCF) produced under varying process parameters, visualized using a stereoscopic microscope.

In preliminary experiments, intact BC pellicles subjected to purification and direct dehydration were evaluated as potential film-forming materials. However, the resulting films were excessively stiff and brittle, making them susceptible to cracking during handling. Similarly, freeze-dried BC pellicles exhibited limited flexibility (Figure 2). These observations prompted the development of a two-step homogenization–dehydration process to obtain BCF with improved macroscopic uniformity and flexibility.

Three preparation parameters were evaluated: the BC-to-water mass ratio, the amount of wet pulp deposited per dish, and the drying temperature. All three parameters influenced the macroscopic characteristics of the resulting BCF (Figure 2). The BC-to-water mass ratio and the amount of wet pulp deposited per dish affected the appearance and handling characteristics of the films. Depending on the preparation conditions, differences in surface uniformity and flexibility were observed, which were considered when selecting the most suitable conditions for subsequent wax coating.

Drying temperature also influenced the macroscopic characteristics of the films. The investigated temperature range (60–80 °C) was selected to evaluate the effects of thermal dehydration on film formation, surface uniformity, and flexibility. Previous studies have demonstrated that drying conditions can significantly affect BC morphology and crystallinity [22], while increasing the drying temperature can accelerate water removal and influence drying kinetics [23]. Furthermore, dehydration may induce structural rearrangements within the cellulose nanofibrillar network, potentially affecting the properties of the resulting material [24]. In the present study, drying at 70 and 80 °C produced visibly more wrinkled and rigid films than drying at 60 °C (Figure S1), suggesting that higher drying temperatures were less favorable for obtaining uniform and flexible BCF under the investigated conditions.

Based on the macromorphological assessment of the investigated preparation parameters, the most suitable conditions among those tested were identified as a BC-to-water mass ratio of 1:2, a wet pulp mass of 45 g per dish, and a drying temperature of 60 °C for 24 h (Figure 2). These conditions yielded relatively smooth, homogeneous, and flexible BCF suitable for subsequent wax coating. The selection was based primarily on macroscopic appearance and handling characteristics rather than a quantitative optimization of mechanical properties.

### 3.2. Optimization of BCF Coating Process

Natural waxes are increasingly used in food packaging for their biodegradable, non-toxic, and moisture-resistant properties, which can improve product appearance, extend shelf life, and reduce moisture loss [25]. Examples such as PBW, CaW, CrW are recognized food additives (E901, E902, E903). Since wax alone does not form durable structures, it is often applied to or combined with polysaccharide films to improve mechanical and barrier performance [26]. In the subsequent stage, the BCF coating process was optimized using twelve natural waxes, ten of plant origin and two derived from beeswax, chosen for their distinct structural characteristics, coloration, and melting points (Table 1). The amount of wax required to obtain visually continuous coverage of the BCF surfaces varied among the investigated formulations, ranging from 4.10 to 6.22 mg/cm^2^, with a mean value of 5.50 mg/cm^2^ (Table 1). The lowest wax loading was recorded for BCF-CaW and BCF-CrW (4.10 mg/cm^2^). These differences may be associated with variations in the physical characteristics of the wax formulations, including their consistency, melting behavior, and spreading properties. However, the individual contributions of these factors were not systematically investigated. Furthermore, the reported wax loading values represent the amount of material applied to obtain visually continuous coverage rather than a quantitative measure of coating efficiency or surface coverage.

### 3.3. Macromorphological Structure of BCF and BCF-W

**Figure 3:**
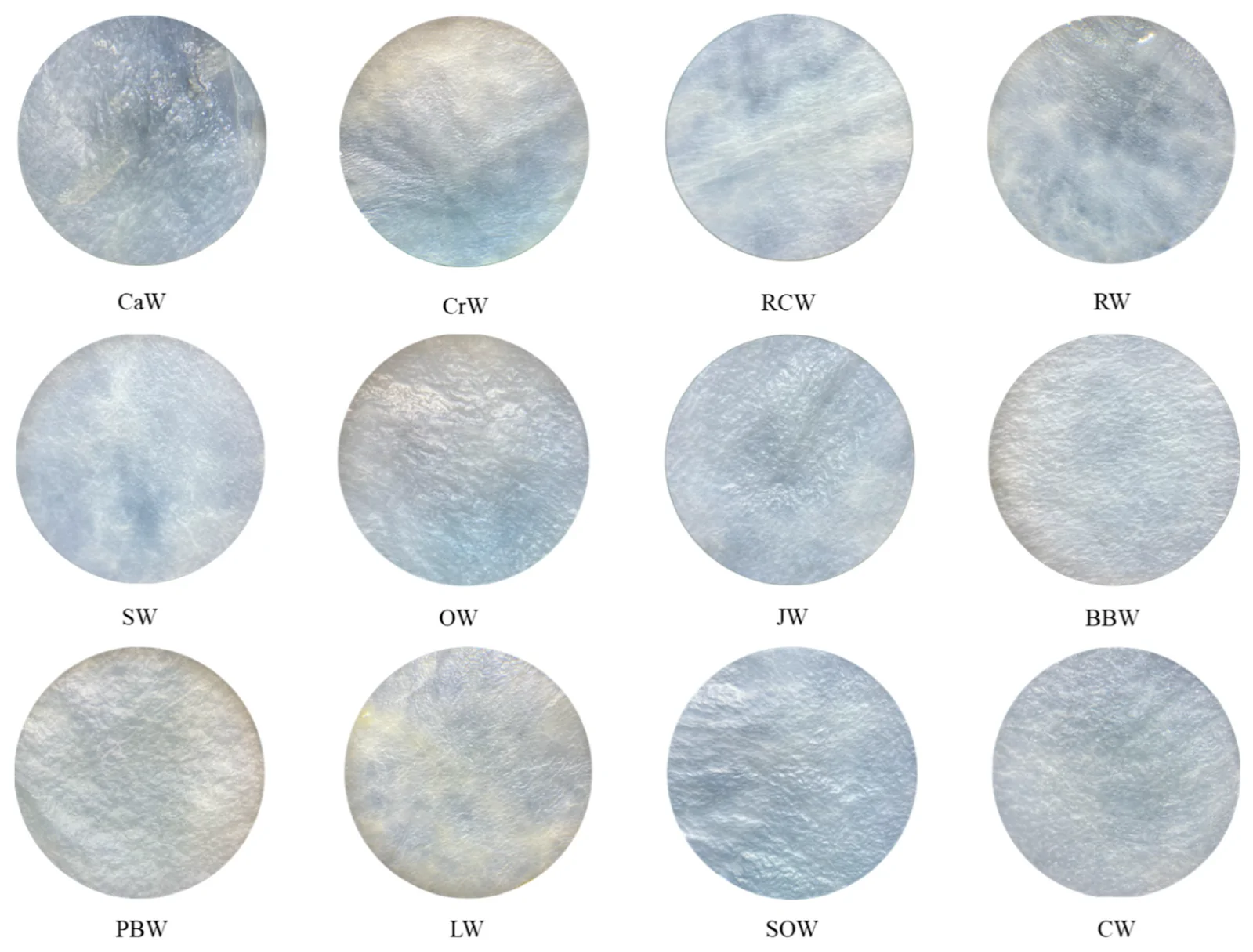
Macromorphological appearance of BCF coated with waxes showing variations in surface characteristics depending on the wax type; candelilla wax (CaW), carnauba wax (CrW), rapeseed-coconut waxes blend (RCW), rapeseed wax (RW), soy wax (SW), olive wax (OW), jojoba wax (JW), bleached beeswax (BBW), pure beeswax (PBW), laurel wax (LW), coconut wax (CW), sunflower-olive waxes blend (SOW).

The effect of wax coating on the macroscopic surface characteristics of BCF was examined using stereoscopic microscopy. Depending on the wax formulation, the resulting BCF-W exhibited differences in coloration, surface appearance, and coating uniformity (Figure 3). Waxes with relatively low melting temperatures (below 50 °C) and a soft, buttery consistency, such as LW, CW, and SOW, were easier to spread during application but remained relatively soft after cooling. During handling, these coatings exhibited visible smearing and localized wax accumulation, resulting in a less uniform surface appearance. In contrast, several wax formulations with higher melting temperatures were more difficult to spread but visually produced more uniform coatings that maintained their appearance during handling. Representative differences in wax spreading are illustrated in Figure S2. These observations suggest that the physical characteristics of the investigated wax formulations influenced their application behavior and the macroscopic appearance of the resulting coatings. However, the present qualitative assessment does not allow coating uniformity or wax–BC interfacial adhesion strength to be quantified.

The behavior of BCF-W during manual application to curved surfaces also varied depending on the wax formulation (Figure S3). BCF-PBW, BCF-RW, BCF-LW, and BCF-RCW exhibited favorable conformability, remaining in contact with the curved surfaces without visible cracking or detachment. In contrast, BCF-CaW, BCF-CrW, BCF-JW, BCF-OW, and BCF-SOW exhibited visible cracking and/or detachment during application. These observations indicate differences in the handling and conformability of the films under the investigated conditions. However, this qualitative assessment does not provide a quantitative measure of flexibility or interfacial adhesion between the wax coatings and the BC substrate.

### 3.4. Water-Related Properties of BCF-W

**Figure 4:**
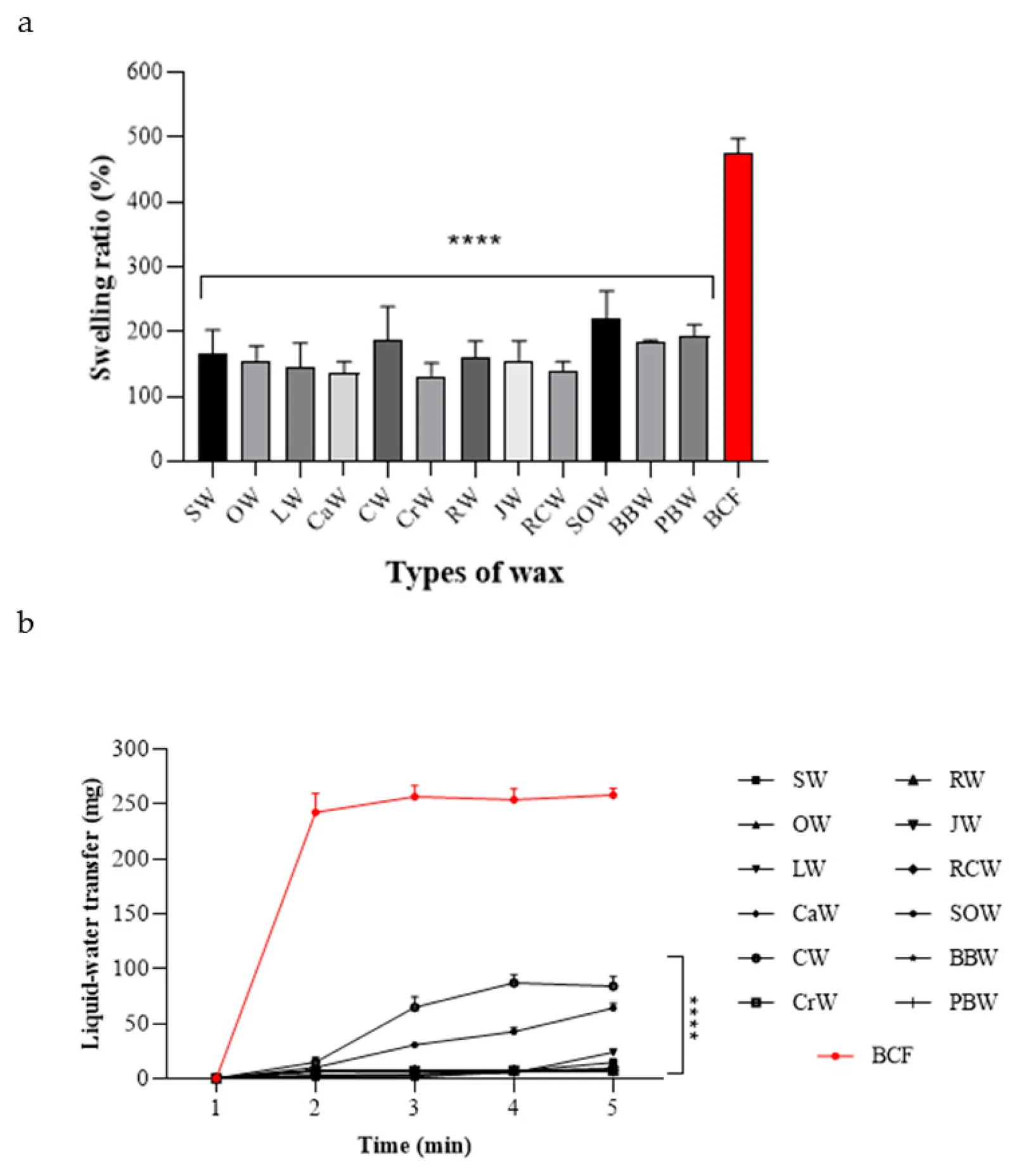
Water-related properties of bacterial cellulose films and wax-coated bacterial cellulose films: (**a**) swelling ratio (*SR*, %) and (**b**) liquid-water transfer (*WT*, mg) over time. Data are presented as mean ± standard error of the mean (SEM). Asterisks (*) denote statistically significant differences between BCF-W and the BCF control: *p* < 0.0001 (****).

Wax coating substantially reduced water-induced swelling and liquid-water transfer compared with uncoated BCF, indicating improved resistance to direct water exposure under the investigated experimental conditions. As shown in Figure 4a, all BCF-W exhibited significantly lower (about threefold) *SR* values than uncoated BCF. Overall, the *SR*% results demonstrate that wax coating reduced water uptake and swelling of BCF, indicating improved resistance to moisture absorption. *SR*% is an important parameter for food packaging applications because excessive water uptake may compromise film dimensional stability and barrier performance during contact with moist food. Therefore, the observed reduction in swelling suggests that wax coating could enhance the suitability of BCF for packaging applications where limiting moisture absorption is essential.

The *WT* results (Figure 4b) demonstrated that uncoated BCF exhibited the greatest amount of water collected by the underlying absorbent paper, whereas most wax-coated formulations substantially reduced (about threefold) water transfer under the investigated conditions. The magnitude of this effect depended on the wax formulation, with BCF-CaW, BCF-CrW, and BCF-RCW exhibiting lower liquid-water transfer values. In contrast, BCF-SOW and BCF-CW showed relatively higher liquid-water transfer and swelling values, although these differences among the wax-coated formulations were not statistically significant. Therefore, the observed numerical trends should not be interpreted as evidence of superior water resistance of any particular wax formulation. These trends may be associated with differences in coating characteristics, including the consistency and surface distribution of the applied waxes, but these potential relationships were not directly investigated.

Contact angle measurements demonstrated a marked increase in surface water repellency following wax coating. The water contact angles of BCF-W ranged from 90.0° to 97.9° (mean: 94.3°), compared with 47.6° for uncoated BCF (Table 2), indicating a pronounced shift toward hydrophobic surface behavior. Although a contact angle of 90° is conventionally used to distinguish hydrophilic from hydrophobic surfaces [27], wettability changes continuously, and values close to this boundary should not be interpreted as indicating an abrupt change in surface properties.

These results align with reports that wax coatings can create superhydrophobic surfaces, reducing moisture uptake and preserving structural integrity [28,29,30]. In turn, improved barrier performance in our study indicates that wax-coating BCF could help maintain food texture, prevent moisture-induced spoilage, and extend shelf life.

Water vapor transmission rate (WVTR) and water vapor permeability (WVP) are also important parameters for assessing the moisture barrier performance of food packaging materials. Previous studies have shown that incorporating hydrophobic waxes into biopolymer-based films and coatings can reduce water vapor transport [31,32]. In particular, cellulose-based multilayer materials containing carnauba wax have been reported to exhibit improved water vapor barrier properties [31]. However, the gravimetric liquid-water transfer assay used in the present study differs fundamentally from standardized WVTR measurements, as it assesses the mass of liquid water collected under specific experimental conditions rather than water vapor transport through the material. Therefore, WVTR measurements under controlled temperature and relative humidity conditions are needed to further evaluate the moisture barrier performance of the developed BCF-W and enable direct comparisons with conventional food packaging materials.

### 3.5. Antimicrobial and Anti-Biofilm Properties of BCF-W

#### 3.5.1. Antimicrobial Properties

According to WHO, 600 million people contract foodborne illnesses annually, with 420,000 deaths [33]. *E. coli*, particularly pathogenic strains like O157:H7, can cause severe illness and is linked to contaminated meat (especially beef) and vegetable crops [34]. *S. aureus* causes approximately 241,000 cases of food poisoning each year in the United States [35], producing heat-stable enterotoxins. Opportunistic fungi such as *C. albicans* can also contaminate food and pose risks to vulnerable populations [36]. The growing demand for antibacterial food packaging reflects the need to reduce such hazards. Antimicrobial packaging incorporates agents into packaging systems to inhibit microbial growth and extend shelf life. These agents, natural or synthetic, may be embedded within the packaging matrix or applied as coatings [37]. Examples include organic acids (e.g., propionic [38], citric [39]), essential oils (thyme, oregano, cinnamon [40]), metal nanoparticles (silver, copper, zinc oxide [41]), and antimicrobial enzymes such as lysozyme [42] and nisin [43]. Chitosan, a biodegradable biopolymer, has also attracted interest for its antimicrobial efficacy [44]. Other strategies involve antimicrobial peptides [45] or bacteriophages [46] that target specific pathogens.

**Figure 5:**
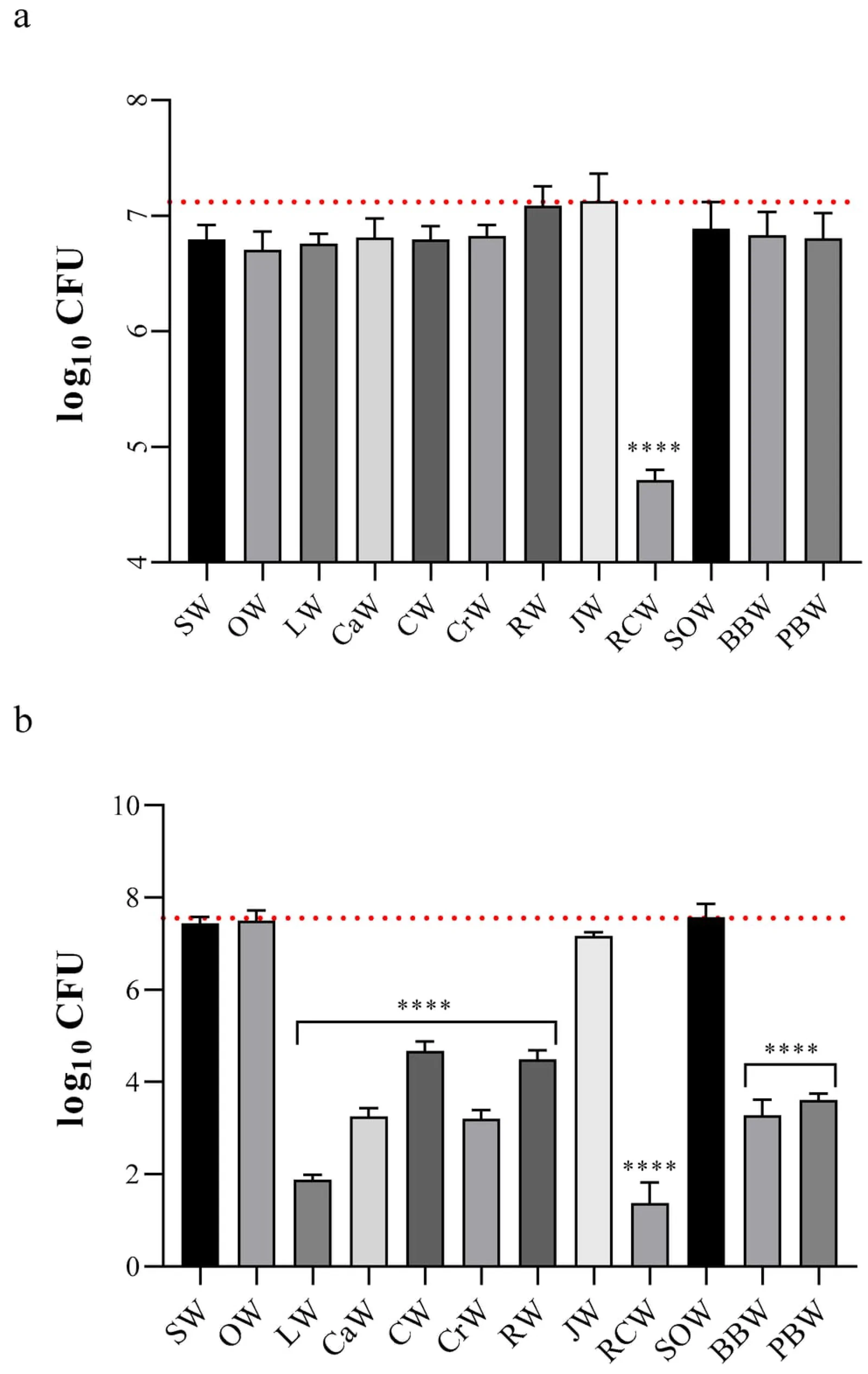

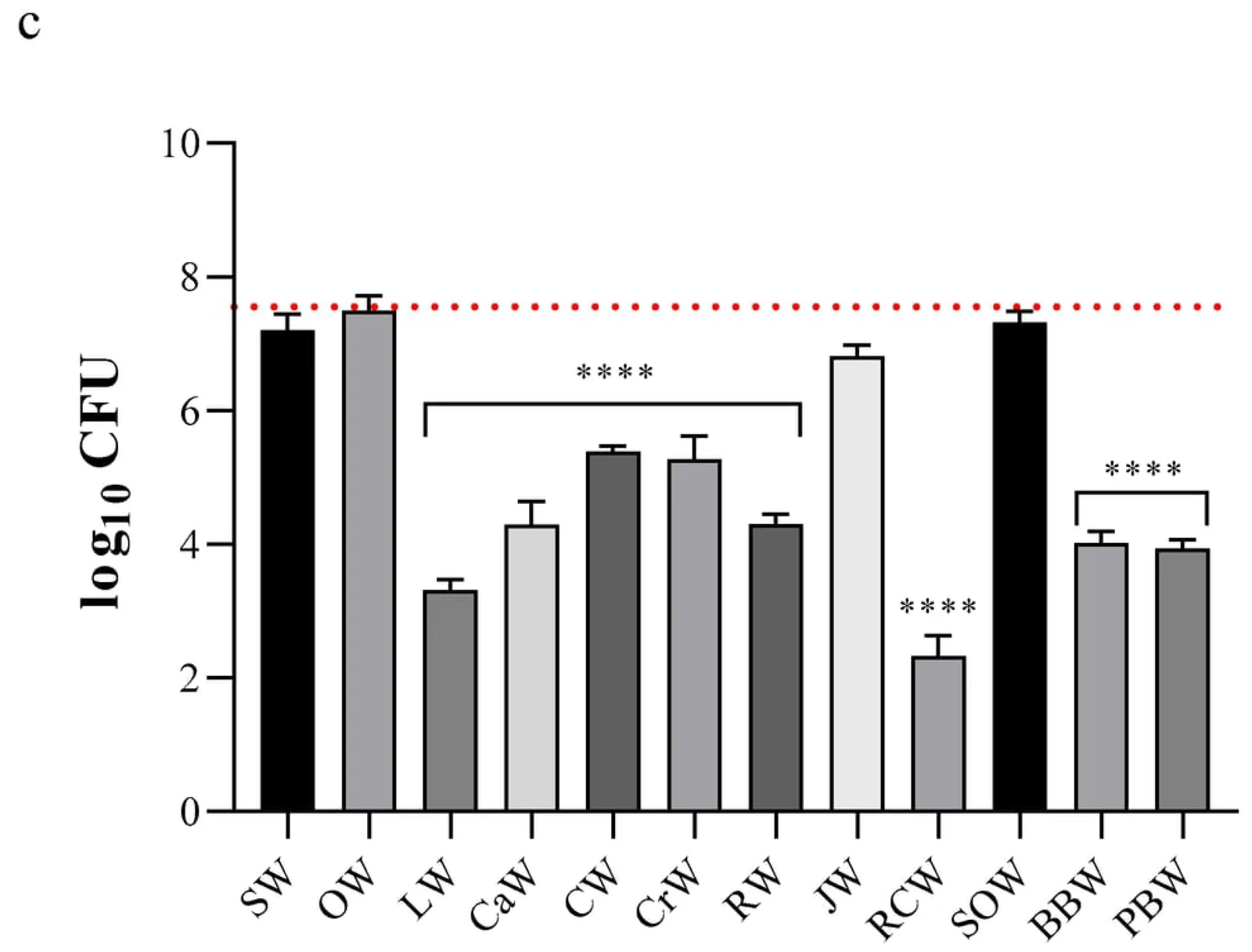
Antimicrobial activity of bacterial cellulose film coated with wax (BCF-W) against (**a**) *E. coli*, (**b**) *S. aureus*, and (**c**) *C. albicans*. Data are presented as mean ± standard error of the mean (SEM). The red line indicates the value for the control bacterial cellulose film (BCF). Asterisks (*) denote statistically significant differences between BCF-W and the BCF control: *p* < 0.0001 (****).

Wax-coated antimicrobial packaging represents another potential approach. The results showed that eight of the investigated wax-coated BCF formulations exhibited statistically significant reductions in viable microbial counts for at least one of the tested microorganisms, with the magnitude of the effect depending on the wax formulation and microbial species (Table S2). Overall, *E. coli* (Figure 5a) exhibited lower susceptibility to the investigated coatings than *S. aureus* (Figure 5b) and *C. albicans* (Figure 5c). For *E. coli*, only BCF-RCW produced a statistically significant reduction in viable cell counts (>99%) compared with uncoated BCF. BCF-RCW also exhibited the greatest observed reductions against S. aureus and *C. albicans*. In addition to BCF-RCW, statistically significant reductions in viable counts of *S. aureus* and *C. albicans* were observed for BCF-LW, BCF-CaW, BCF-CW, BCF-CrW, BCF-RW, BCF-BBW, and BCF-PBW (>99%). In contrast, BCF-SW, BCF-OW, BCF-JW, and BCF-SOW did not produce statistically significant reductions in viable microbial counts under the investigated conditions.

Although several wax coatings exhibited statistically significant antimicrobial effects, the magnitude of these reductions varied considerably, and the observed activity should not be interpreted as uniformly strong across all tested microorganisms. The lower susceptibility of *E. coli* may be attributed to its Gram-negative outer membrane, whose lipopolysaccharide-rich outer leaflet restricts the penetration of many hydrophobic compounds. Furthermore, outer membrane porins primarily facilitate the transport of small hydrophilic molecules, potentially limiting the accessibility of bioactive wax constituents to bacterial cells [47,48]. Previous studies have associated antimicrobial properties of certain natural waxes and their constituents with fatty acids, esters, terpenoids, and other bioactive compounds. The lauric acid, the predominant medium-chain fatty acid in CW, has been reported to exhibit antimicrobial activity, potentially through interactions with microbial cell membranes and disruption of membrane integrity [49,50]. PBW and BBW are complex mixtures of long-chain fatty acid esters, free fatty acids, hydrocarbons, and minor constituents whose proportions may vary depending on the origin and processing of the raw material. Their antimicrobial activity has been reported against selected Gram-positive and Gram-negative bacteria, yeasts, and filamentous fungi, although the available evidence remains limited and the underlying mechanisms are not fully elucidated [51]. CrW contains an esterified and free phenolic compound, such as p-methoxycinnamic acid, which has been associated with the suppression of fungal and bacterial growth [52]. Moreover, its high hydrophobicity may enable the formation of a protective barrier on food surfaces, limiting moisture transfer and transpiration [53]. Similarly, the primary component of LW, myristic acid, exhibits proven antimicrobial and antifungal activities, particularly against Gram-positive bacteria and *C. albicans*, by effectively disrupting pathogen cell membranes [54,55]. In contrast, CaW and RW can provide antimicrobial protection predominantly through passive physical mechanisms that impair pathogen adhesion and growth. The high-molecular-weight n-alkanes in CaW are proven to assemble into a dense, hydrophobic crystalline matrix that severely restricts moisture permeability and oxygen diffusion, effectively arresting microbial colonization [56]. Similarly, the high concentration of stearic acid in RW can form a superhydrophobic surface and therefore mitigate initial bacterial and fungal attachment [57]. Notably, the enhanced efficacy of RCW may be attributed to a dual-action mechanism: the high content of antimicrobial lauric acid derived from CW in tandem with the high concentration of superhydrophobic stearic acid from RW.

Although the antimicrobial activity of natural waxes has previously been associated with various lipid constituents and other bioactive compounds, the commercial wax formulations investigated in the present study were not subjected to independent chemical characterization. However, the mentioned bioactive compounds are listed in the technical specifications of the commercial wax formulations. It should also be emphasized that the antimicrobial assay quantified recoverable viable microbial cells following incubation, whereas biofilm-associated biomass was assessed separately using crystal violet staining (Section 3.5.2).

#### 3.5.2. Anti-Biofilm Properties

**Figure 6:**
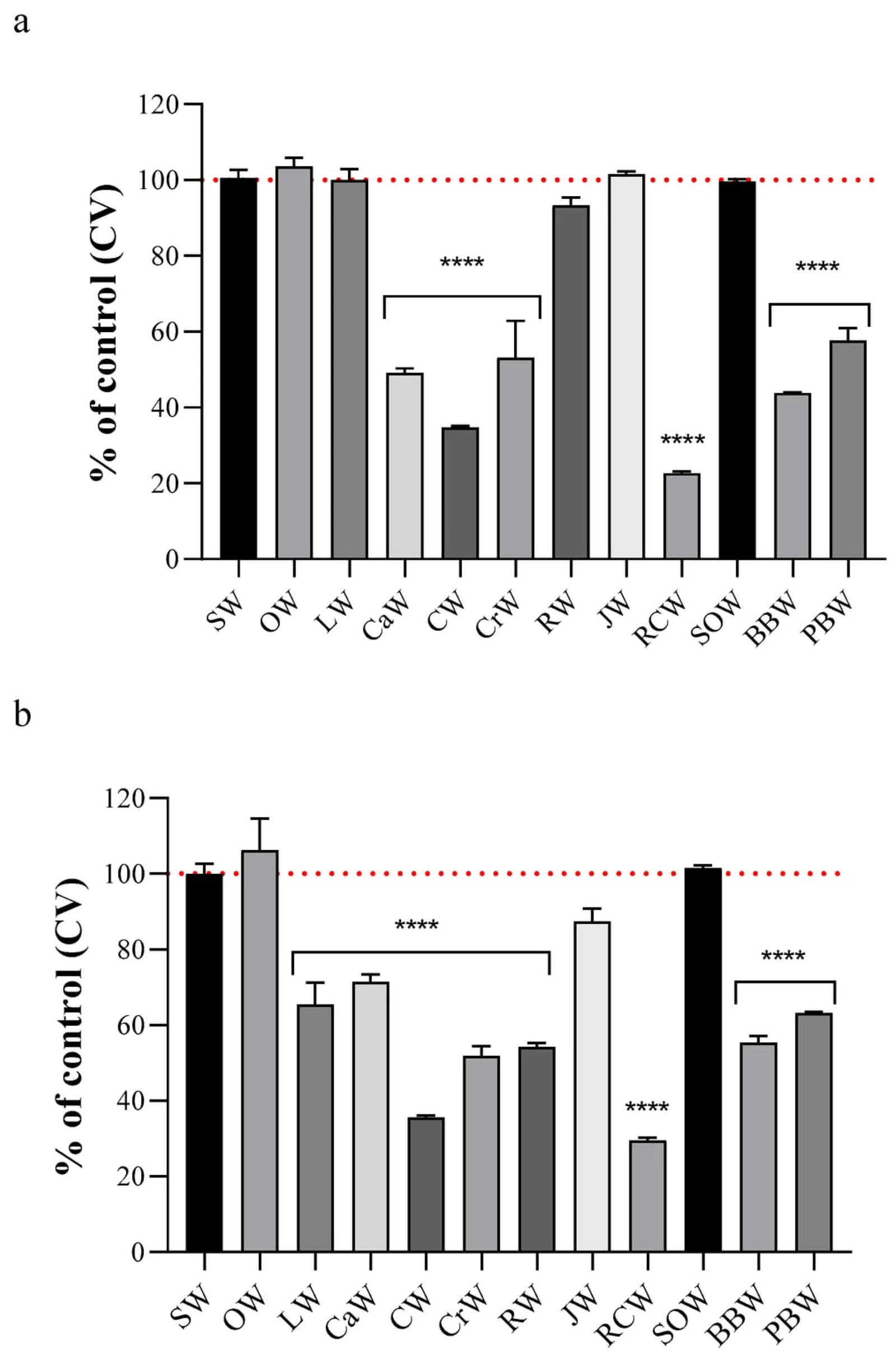

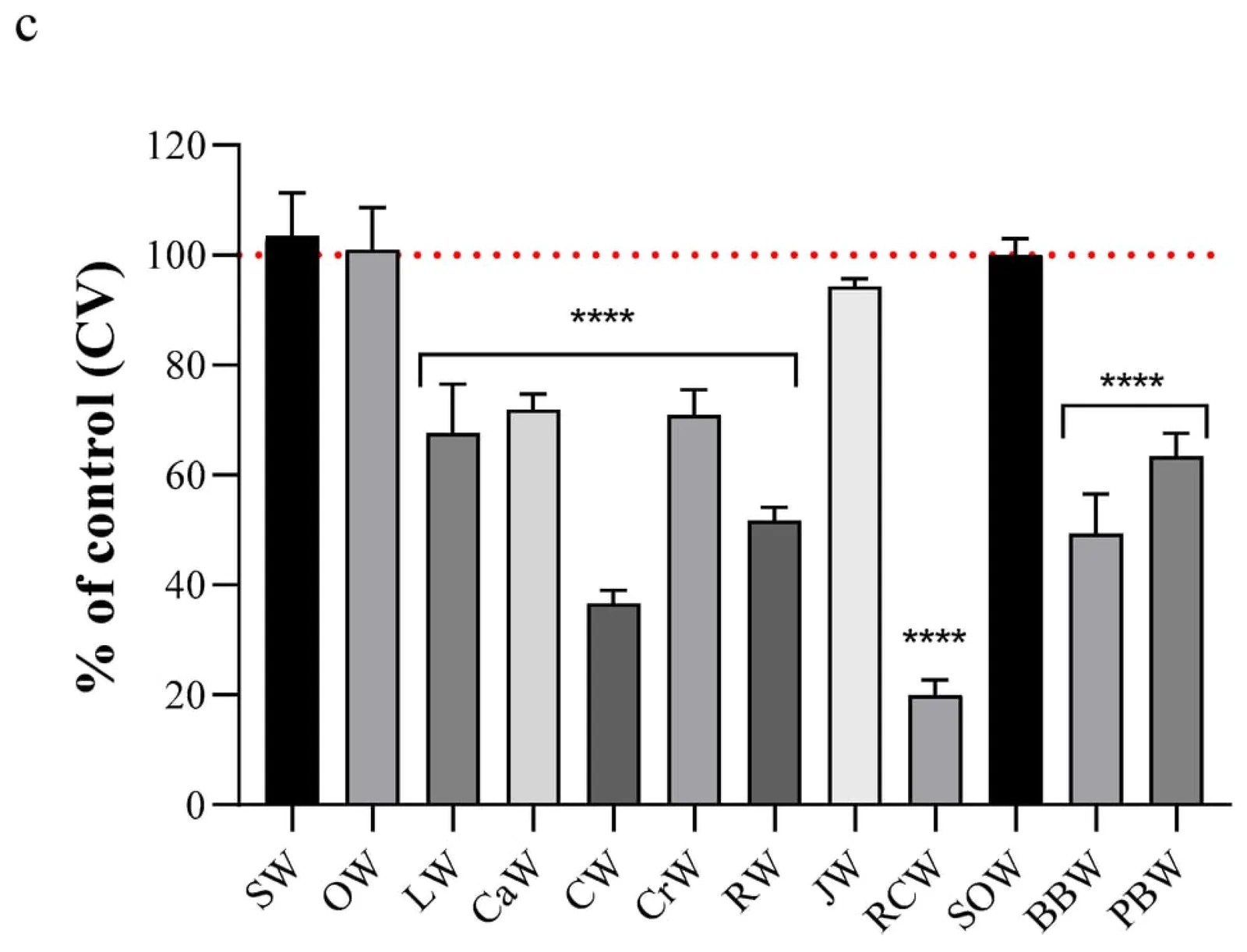
Antibiofilm activity of bacterial cellulose films coated with waxes (BCF-W) against (**a**) *E. coli*, (**b**) *S. aureus*, and (**c**) *C. albicans*. Data are presented as mean ± standard error of the mean (SEM). The red line indicates the value for the control bacterial cellulose film (BCF). Asterisks (*) denote statistically significant differences between BCF-W and the BCF control: *p* < 0.0001 (****).

Several wax-coated BCF formulations exhibited reduced biofilm-associated biomass compared with uncoated BCF, with the magnitude of the effect depending on the wax formulation and the microorganism tested (Figure 6a–c). The most pronounced reductions were observed for selected formulations, including BCF-RCW, BCF-CaW, BCF-CW, BCF-CrW, BCF-RW, BCF-BBW, and BCF-PBW. The mean reductions in biofilm-associated biomass were approximately 33% for *S. aureus* and 31% for *C. albicans* compared with uncoated BCF. The results for these microorganisms correspond to those obtained in antimicrobial studies. However, several BCF-W also exhibited reduced crystal violet staining associated with *E. coli* biofilm formation, although most of these formulations did not produce a statistically significant reduction in recoverable viable *E. coli* counts in the antimicrobial assay. These differences between the antimicrobial and antibiofilm results (Figure 5a and Figure 6a) can be explained, at least in part, by the distinct experimental endpoints and assay conditions. The antimicrobial assay quantified recoverable viable bacterial cells using colony counting, whereas the crystal violet assay estimated biofilm-associated biomass, including bacterial cells and extracellular matrix components, without distinguishing between viable and nonviable cells. Furthermore, the antimicrobial assay involved 0.4 g of material exposed to 200 µL of bacterial suspension, whereas the antibiofilm assay used 0.1 g of material immersed in 2 mL of bacterial suspension. These differences in experimental configuration may contribute to the observed variation in microbial responses. Consequently, reduced crystal violet staining does not necessarily indicate a proportional reduction in viable cell counts. Nevertheless, BCF-RCW exhibited favorable results in both assays, indicating that its effects were not restricted to a single experimental endpoint.

The reduction in biofilm-associated biomass observed for some formulations in the absence of a corresponding antibacterial effect may be associated with changes in microbial attachment or biofilm accumulation on the wax-coated surfaces, as described for, e.g., CaW and RW in the previous section. Such effects may depend on several interrelated surface characteristics, including wettability, roughness, and chemical composition [58]. Although the individual contributions of these factors were not determined in the present study, the results suggest that wax modification may influence biofilm accumulation through surface-related effects in addition to any antimicrobial activity associated with the coating constituents.

### 3.6. Surface Micromorphology and Mechanical Characteristics of BCF and BCF-RCW

Based on its favorable surface, water-related and antimicrobial performance, the BCF-RCW formulation was selected for further advanced analyses, including SEM and mechanical testing. These analyses aimed to provide a more comprehensive understanding of the surface micromorphology and mechanical properties of the selected wax-coated films. As BCF-RCW was subsequently selected for the preliminary proof-of-concept food-storage experiment, further characterization was considered important to better understand its properties and assess its potential suitability for food packaging applications.

**Figure 7:**
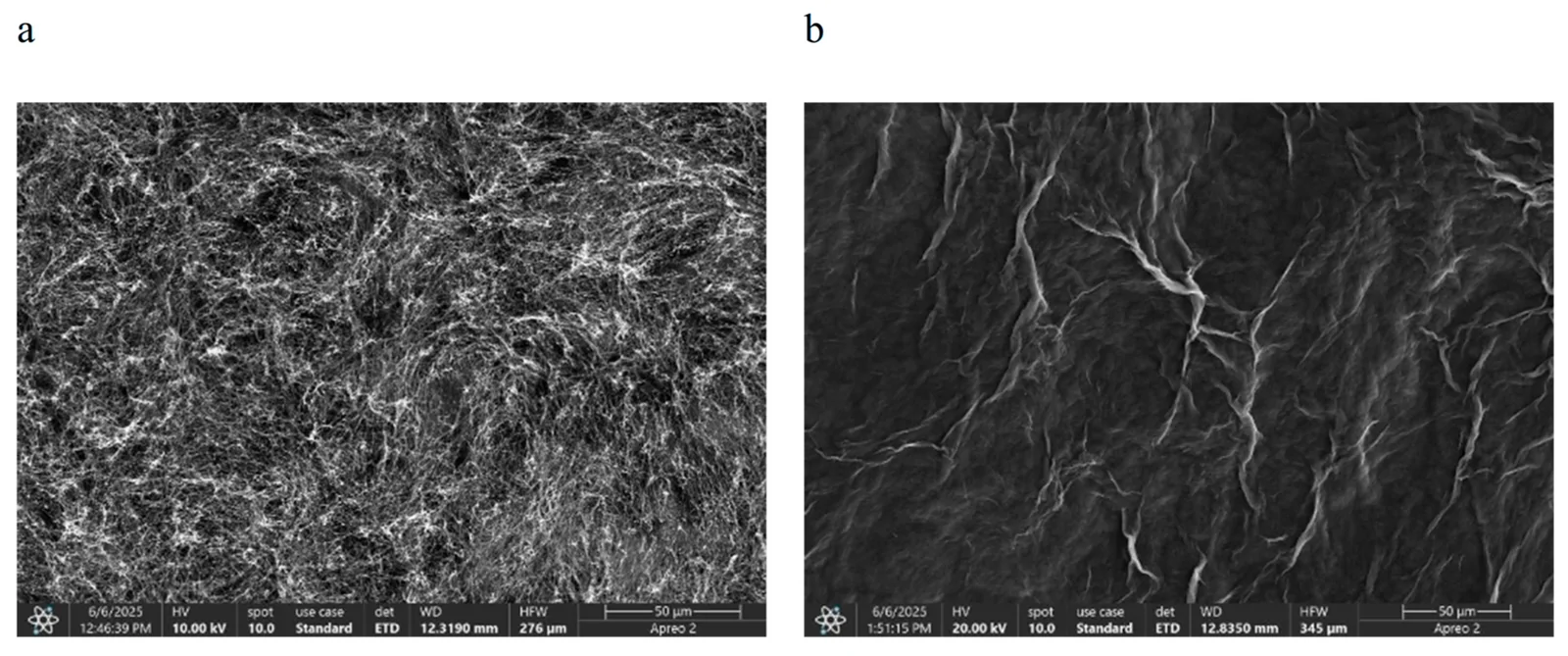
Representative images of (**a**) bacterial cellulose film (BCF) and (**b**) rapeseed-coconut waxes blend coated bacterial cellulose film (BCF-RCW) microstructure, visualized using scanning electron microscopy (SEM).

Scanning electron microscopy revealed distinct differences in the surface morphology of uncoated BCF (Figure 7a) and BCF-RCW (Figure 7b). The uncoated BCF exhibited an interconnected cellulose fibrillar network with a porous surface structure, whereas BCF-RCW displayed a comparatively smooth and continuous surface layer covering much of the underlying cellulose network, consistent with the presence of the applied wax coating. Similar morphological observations were reported by Indriyati et al. [17], who described beeswax covering the BC network and penetrating membrane pores. However, the present SEM observations are limited to surface morphology and do not establish the extent of wax penetration into the BCF structure, the uniformity of the coating throughout the material, or the strength of wax–BC interfacial adhesion.

Although electron-beam exposure may induce localized heating and affect the morphology of thermally sensitive wax coatings, no conclusions regarding such alterations can be drawn from the present observations. Nevertheless, the SEM images provide useful qualitative evidence of the morphological changes associated with wax coating, although they do not allow the extent of wax penetration or the strength of wax–BC interfacial adhesion to be determined.

Table 3 summarizes the mechanical properties of uncoated BCF and BCF-RCW. Wax coating increased the mean film thickness from 0.12 ± 0.04 mm to 0.23 ± 0.05 mm. In contrast, the mean maximum load decreased from 88.0 ± 24.0 N to 74.7 ± 24.8 N. More pronounced reductions were observed in the nominal stress-based mechanical parameters. Tensile strength decreased from 32.0 ± 12.7 MPa to 14.8 ± 7.7 MPa, while stress at break decreased from 31.4 ± 13.3 MPa to 14.6 ± 7.77 MPa. Similarly, the modulus determined within the 0.05–0.25% strain range decreased from 485 ± 225 MPa to 256 ± 68 MPa, and the automatically determined modulus decreased from 968 ± 372 MPa to 361 ± 157 MPa. Both elongation parameters were numerically higher in BCF-RCW than in uncoated BCF.

The differences in film thickness are particularly relevant to the interpretation of these results. The additional wax layer increases the total cross-sectional area of the composite film without necessarily providing a proportional increase in its load-bearing capacity. This interpretation is consistent with the relatively small difference in mean maximum load compared with the more pronounced reduction in nominal tensile strength. Therefore, the lower stress-based mechanical parameters of BCF-RCW should not be interpreted solely as evidence of deterioration of the underlying cellulose network. The measured mechanical response reflects the composite’s properties, including contributions from the coating and potential structural differences between the films. A similar reduction in tensile strength accompanied by increased elongation at break was reported by Indriyati et al. [17] following beeswax incorporation into BC-based films.

### 3.7. Preliminary Proof-of-Concept Food-Storage Experiment

**Figure 8:**
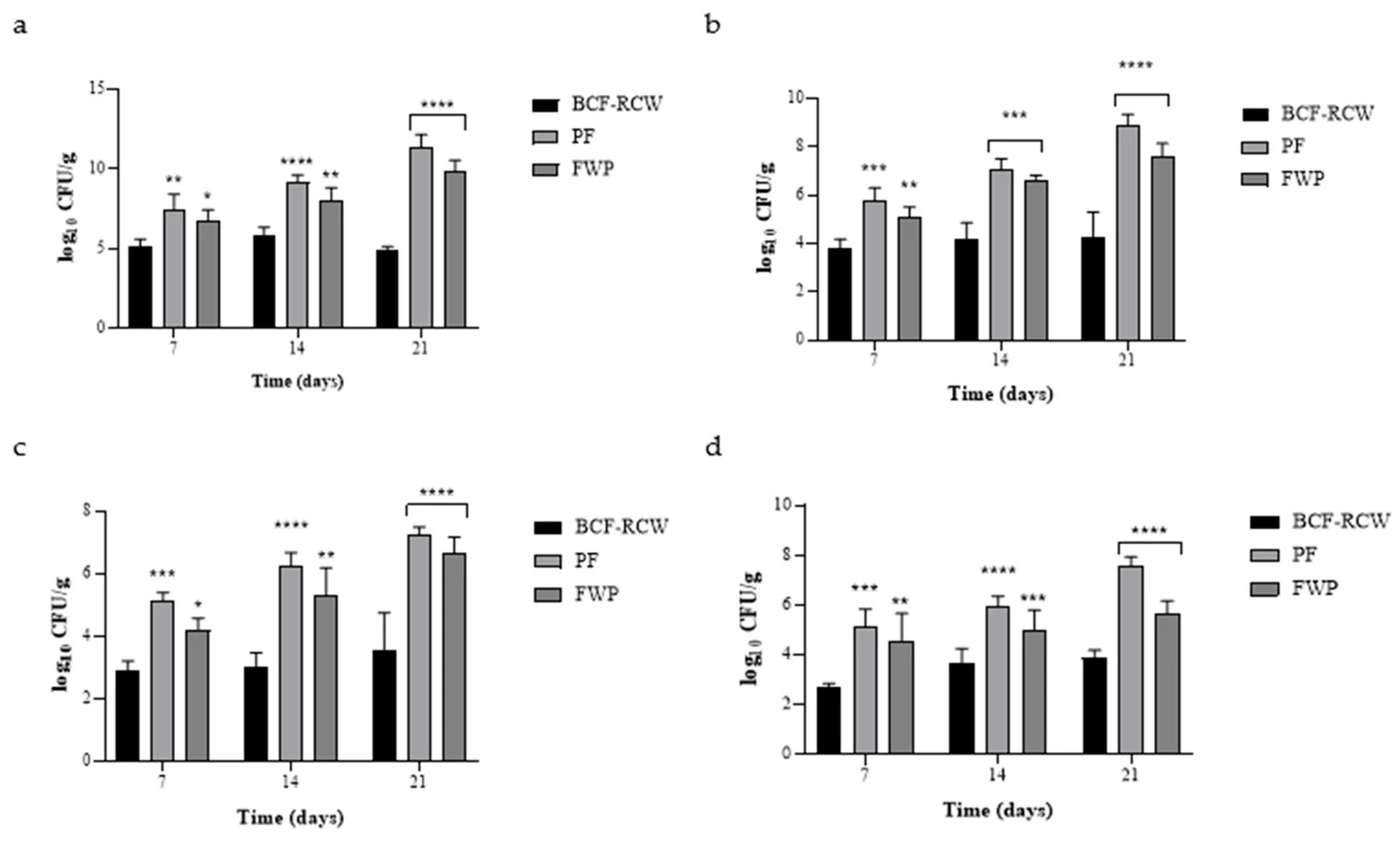
The number of microorganisms on food samples stored using bacterial cellulose films coated with rapeseed-coconut waxes blend (BCF-RCW), polyethylene film (PF) and food wrapping paper (FWP): (**a**) cheese, (**b**) bread, (**c**) apple, (**d**) ham. Data are presented as mean ± standard error of the mean (SEM). Asterisks (*) indicate statistics significant differences between the indicated packaging groups: *p* < 0.05 (*), *p* < 0.01 (**), *p* < 0.001 (***), and *p* < 0.0001 (****).

The preliminary food-storage experiment provided an initial assessment of the potential of BCF-RCW as a food-packaging material under refrigerated conditions. As shown in Figure 8a–d, food samples wrapped in BCF-RCW generally exhibited lower recoverable culturable microbial counts than those wrapped in polyethylene film (PF) or food wrapping paper (FWP) during the 21-day storage period. The magnitude of these differences varied depending on the food product and storage time.

The average number of microorganisms in the case of BCF-RCW ranged from 3.39 to 5.27 log_10_ CFU/g depending on the food sample. These values did not change significantly during the incubation period. In the case of PF and FWP, a continuous increase in the number of microorganisms was observed during 21 days of incubation for each food sample. Collectively, after 21 days, BCF-RCW produced a statistically significant reduction in viable cell counts (>99%) compared to PF and FWP in all stored food products.

These observations suggest that BCF-RCW may contribute to limiting microbial accumulation on selected food products under the investigated conditions. Nevertheless, the experiment was designed as a preliminary proof-of-concept assessment using small food portions, and further studies under representative packaging and storage conditions are needed to evaluate its practical applicability.

An additional consideration for the application of BCF-W in direct-contact food packaging is their potential influence on the sensory properties of food. Natural waxes differ in chemical composition, and the incorporation of waxes or other lipid-based components into food-contact formulations may affect sensory attributes, including taste and aroma [59,60]. Moreover, the transfer of odor-active compounds from packaging materials to food may depend on the material composition and contact conditions [61]. Although wax-based coatings can improve food preservation, their natural origin does not necessarily guarantee sensory neutrality. Since no formal sensory evaluation or migration testing was performed in the present study, potential changes in food taste and aroma cannot be excluded. Therefore, future studies should assess the sensory compatibility and migration behavior of the most promising BCF-W formulations under relevant food-contact conditions.

## 4. Conclusions

This study investigated the potential of wax-coated bacterial cellulose films (BCF-W) as bio-based materials for food packaging applications through the combination of a two-step homogenization–dehydration process followed by surface coating with commercially available natural wax and wax-like formulations. Process optimization (BC-to-water mass ratio 1:2, with 45 g of pulp deposited in each dish, drying at 60 °C) yielded relatively smooth, homogeneous, and flexible BCF, suitable for wax coating. Compared to uncoated BCF, wax-coated films exhibited reduced water-induced swelling and liquid-water transfer, together with increased surface water repellency, as indicated by water contact angles ranging from 90.0° to 97.9°. These findings suggest improved resistance to liquid-water exposure under the investigated experimental conditions. The antimicrobial evaluation revealed formulation-dependent effects, with several wax-coated films, including BCF-RCW, BCF-LW, BCF-CaW, BCF-CW, BCF-CrW, BCF-RW, BCF-BBW, and BCF-PBW, exhibiting statistically significant reductions in viable counts of *S. aureus* and *C. albicans*. Among the investigated formulations, BCF-RCW was the only one showing a statistically significant reduction in viable *E. coli* counts. The biofilm assays also indicated that some wax-coated formulations reduced biofilm formation, including formulations that did not exhibit significant antimicrobial activity. These observations suggest that wax coatings may influence microbial colonization through mechanisms not directly associated with reductions in cell viability, although the underlying mechanisms require further investigation. Based on total best performance, BCF-RCW was selected for a preliminary refrigerated storage test, in which a marked reduction in viable pathogen counts was observed compared with conventional food packaging materials.

Overall, the results demonstrate that surface modification of BC with selected commercially available wax formulations can improve certain water-related and biological properties relevant to food-packaging applications. However, the biodegradability of the developed BCF-W composites was not directly evaluated and should be confirmed in future studies. Furthermore, additional investigations addressing water vapor barrier properties, long-term food-storage performance, coating stability, regulatory requirements, and industrial scalability are needed to assess their practical applicability. The developed materials therefore represent a promising direction for further research into bio-based food-packaging systems rather than a fully validated alternative to conventional packaging materials.

## Figures and Tables

**Table 1:** Selected physicochemical characteristics of the natural waxes used for bacterial cellulose films (BCF) coating.

Commercial Wax Formulation	Appearance	Biological Origin	Physical Form	Density [g/cm^3^]	Melting Range [°C]	Wax Loading [mg/cm^2^]
Candelilla (CaW)	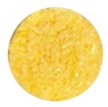	*Euphorbia antisyphilitica* leaves	Flakes	0.95	68–73	4.10
Carnauba (CrW)	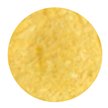	*Copernica cerifera* leaves	Flakes	0.99	80–88	4.10
Rapeseed- coconut (RCW)	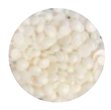	*Brassica napus* seeds and *Cocos nucifera* nut pulp	Granules	0.92	46–55	5.94
Rapeseed (RW)	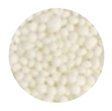	*Brassica napus* seeds	Granules	0.99	65–75	6.22
Soy (SW)	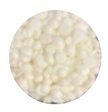	*Glycine max* grains	Granules	0.90	51–54	5.94
Olive (OW)	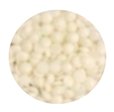	*Olea europaea* fruit pulp	Granules	0.92	60–62	5.94
Jojoba (JW)	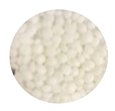	*Simmondsia chinensis* seeds	Granules	0.95	60–70	6.22
Beeswax bleached (BBW)	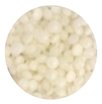	Product of honeybees (*Apis mellifera* and *Apis cerana*)	Granules	0.96	64–65	5.66
Pure beeswax (PBW)	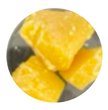		Solid (hard)	0.92	64–66	5.65
Laurel (LW)	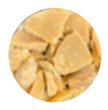	*Myrica pubescens* fruits	Solid (hard)	0.96	35–45	4.38
Sunflower- olive (SOW)	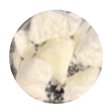	*Helianthus annuus* seeds and *Olea europaea* fruit pulp	Solid (buttery)	0.92	44–48	5.94
Coconut (CW)	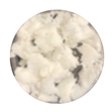	*Cocos nucifera* nut pulp	Solid (buttery)	0.95	31–35	5.94

Note: The physical form, density, and melting ranges were obtained from the supplier’s technical specifications and were not independently determined in this study. Wax loading was determined experimentally during the coating procedure. Additional supplier-reported physicochemical and compositional information is provided in Supplementary Table S1. The commercial designation “wax” does not necessarily indicate a chemically pure wax, and the complete composition of certain formulations, including possible additional ingredients, was not available.

**Table 2:** Water contact angle measurements of BCF and BCF-W.

BCF-W												BCF
SW	OW	LW	CaW	CW	CrW	RW	JW	RCW	SOW	BBW	PBW	
90.0	95.4	97.3	91.4	92.5	93.4	97.9	93.8	94.6	92.8	95.8	95.6	47.6

Note: A contact angle of 90° is conventionally used as a reference for distinguishing hydrophilic and hydrophobic surface behavior. Values close to this boundary do not necessarily indicate substantial differences in wettability.

**Table 3:** Mechanical properties of bacterial cellulose film (BCF) and bacterial cellulose films coated with waxes (BCF-W).

Analyzed Sample	Width	Thickness	Max Load	Tensile Strength	Elongation at Max Stress	Stress at Break	Elongation at Break	Modulus 0.05–0.25%	Automatic Modulus
	(mm)	(mm)	(N)	(MPa)	(%)	(MPa)	(%)	(MPa)	(MPa)
BCF	25.71 ± 0.75	0.12 ± 0.04	88.0 ± 24.0	32.0 ± 12.7	4.62 ± 0.70	31.4 ± 13.3	4.68 ± 0.70	485 ± 225	968 ± 372
BCF-W	23.52 ± 2.38	0.23 ± 0.05	74.7 ± 24.8	14.8 ± 7.7	5.95 ± 2.39	14.6 ± 7.77	5.99 ± 2.39	256 ± 68	361 ± 157
*p*-value	**0.029**	**<0.001**	0.220	**0.010**	*0.062*	**0.015**	*0.067*	**0.036**	**0.004**

Values are presented as mean ± standard deviation. *p*-values indicate comparisons between uncoated bacterial cellulose films (BCF) and wax-coated bacterial cellulose films (BCF-W). Differences were considered statistically significant at *p* < 0.05 and bolded. Italicized *p*-values indicate a tendency towards a difference that did not reach statistical significance (0.05 ≤ *p* < 0.10).

## Data Availability

The data that support the findings of this study are available from the corresponding author upon reasonable request.

## Supplementary Materials

The following supporting information can be downloaded at: https://www.mdpi.com/article/10.3390/polym18212705/s1, Table S1. Physicochemical characteristics and chemical composition of natural waxes and wax blends used for bacterial cellulose film modification; Table S2. Antimicrobial activity of wax-coated bacterial cellulose films against selected microorganisms; Figure S1. Macroscopic appearance of bacterial cellulose films (BCF) prepared at different drying temperatures: (a) 60 °C, (b) 70 °C, and (c) 80 °C. Images were acquired using a stereomicroscope; Figure S2. Representative differences in the surface spreading of wax formulations with different melting temperature ranges on bacterial cellulose films (BCF): (a) coconut wax (CW; 31–35 °C) and (b) rapeseed wax (RW; 65–75 °C); Figure S3. Qualitative assessment of the conformability of bacterial cellulose films coated with twelve commercial wax formulations (BCF-W) during manual application. Wax formulation abbreviations are defined in Table 1.
