# Supplementary material for: Wax-Functionalized Bacterial Cellulose Foils as Renewable Food Packaging with Enhanced Barrier and Antimicrobial Performance
Source: Polymers (Basel). 2026 Nov 1;18(21):2705. doi: 10.3390/polym18212705 (PMC13765732; doi:10.3390/polym18212705)
Supplement: Supplementary file 1 [file polymers-18-02705-s001.zip › polymers-4620005-supplementary.pdf]

Supplementary Materials

Table S1. Physicochemical characteristics and chemical composition of natural waxes and wax blends used for bacterial cellulose film modification.

Wax	Acid value [mg KOH/g]	Needle penetration [d-mm in 25°C]	Ester value [mg KOH/g]	Peroxide value [meq O_2/kg]	Saponificat ion value [mg KOH/g]	Chemical composition
**Candelilla (CaW)**	12–18	0-2	33	0	43-65	Mainly alkanes; High-molecular-weight esters; Alcohols and sterols (20.0-29.0%); Free fatty acids (7.0-9.0%); Resins (12.0-14.0%).
**Carnauba (CrW)**	2-7	1.25	71-88	<5	79-95	Esters (80.0%) including aliphatic esters and cinnamic acid diesters; Monohydric alcohols; Fatty acids, including tetracosanoic acid; Aromatic acids, including p-hydroxycinnamic acid and p-methoxycinnamic acid, mainly in the form of oligomers and po lymers.
**Rapeseed–coconut (RCW)**	No data available	30	No data available	<2	No data available	The mixing ratio of the rapeseed–coconut wax blend (RCW) was 70% rapeseed wax and 30% coconut wax. Mainly trialkylglycerides (triglycerides of C8-C18 saturated and C18 unsaturated fatty acids); Mono- and dialkylglycerides; Free fatty acids (max 0.5%).
**Rapeseed (RW)**	16.5	20	163.5-183.5	<1	180-200	Fatty acid composition: Palmitic acid (4.9%); Stearic acid (92.1%); Arachidic acid (1.9%); Behenic acid (0.5%).
**Soy (SW)**	<3	21	187-217	<4	190-220	Fatty acid composition: Myristic acid (0.5%); Palmitic acid (9.0-16.0%); Stearic acid (79.0-89.0%); Oleic acid (≤ 4.0%); Linoleic acid (≤ 1.0%); α-Linolenic acid (≤ 0.2%);

						Arachidic acid (≤ 1.0%); Behenic acid (≤ 1.0%).
**Olive (OW)**	2	23	173-198	No data available	175-200	Olive-derived lipid components, potentially including fatty acid esters and glycerides. The detailed fatty acid profile and proportions of individual constituents were not available for the commercial formulation used in this study.
**Jojoba (JW)**	2	No data available	148-183	2,0	150-185	Fatty acid composition: Palmitic acid (3.0%); Palmitoleic acid (1.0%); Oleic acid (5.0-15.0%); Eicosenoic acid (65.0-80.0%); Behenic acid (1.0%); Erucic acid (10.0-20.0%); Ervonic acid (3.0%).
**Beeswax bleached (BBW)**	17-18.7	22	68-82	<5	87-104	Monoesters (45-55%); Hydrocarbons (15-18%); Free fatty acids (10-15%); Diesters and complex esters (8-12%); Hydroxy monoesters (4-6%); Free fatty alcohols (1-2%).
**Pure beeswax (PBW)**	17-24	22	70-80	<5	87-104	
**Laurel (LW)**	14-17	7-16	193-226	No data available	210-240	Fatty acid composition: Caprylic acid (0.01%); Lauric acid (0.4%); Myristic acid (54.0%); Palmitic acid (42.0%); Stearic acid (0.25%); Arachidic acid (0.04%); Behenic acid (0.03%).
**Sunflower–olive (SOW)**	No data availab le	52	No data available	No data available	No data available	The mixing ratio of the sunflower–olive wax blend (SOW) was 50% sunflower wax and 50% olive wax. The detailed chemical composition was not disclosed i n the available product documentation.
**Coconut (CW)**	0.9	32	249.1-264.1	<1	250-265	Fatty acid composition: Caproic acid (1.5%); Caprylic acid (2.0–11.5%); Capric acid (4.6–11.0%); Lauric acid (40.0–60.0%); Myristic acid (13.0–25.0%);

Palmitic acid (6.0–12.0%);
Stearic acid (1.0–5.0%);
Oleic acid (3.0–12,0%);
Linoleic acid (1.0–3.0%);
Linolenic acid (0.5%);
Arachidic acid (0.5%).

Note: The physicochemical characteristics and compositional information presented in this table were compiled from the available technical specifications of the commercial wax formulations. Values represent supplier-declared specifications rather than independent measurements performed in this study. "Not available" indicates that the corresponding information was not provided in the available documentation. The detailed composition of commercial wax blends may differ from that of their individual raw materials.

Table S2. Antimicrobial activity of wax-coated bacterial cellulose films against selected microorganisms.

Microorganism	Formulation	Viable count (log_{10} CFU), mean ± SEM	Reduction (%)	p-value
E. coli	SW	6.846 ± 0.039	51.39	0.2092
	OW	6.793 ± 0.049	56.95	0.0737
	LW	6.809 ± 0.012	55.29	0.1043
	CaW	6.812 ± 0.082	54.95	0.1114
	CW	6.796 ± 0.057	56.62	0.0791
	CrW	6.824 ± 0.049	53.75	0.1399
	RW	7.090 ± 0.083	14.68	1.0000
	JW	7.129 ± 0.118	6.73	1.0000
	RCW	4.713 ± 0.044	99.64	**<0.0001**
	SOW	6.887 ± 0.117	46.51	0.4042
	BBW	6.831 ± 0.102	53.02	0.1594
	PBW	6.804 ± 0.111	55.86	0.0928
	**BCF**	**7.159 ± 0.036**	—	—
S. aureus	SW	7.437 ± 0.051	23.79	0.9991
	OW	7.463 ± 0.087	19.21	0.9999
	LW	1.880 ± 0.035	>99.99	**<0.0001**
	CaW	3.208 ± 0.055	>99.99	**<0.0001**
	CW	4.673 ± 0.077	99.87	**<0.0001**
	CrW	3.201 ± 0.073	>99.99	**<0.0001**
	RW	4.488 ± 0.087	99.91	**<0.0001**
	JW	7.160 ± 0.030	59.77	0.1057
	RCW	1.308 ± 0.169	>99.99	**<0.0001**
	SOW	7.531 ± 0.113	5.48	1.0000
	BBW	3.321 ± 0.134	>99.99	**<0.0001**
	PBW	3.614 ± 0.050	99.99	**<0.0001**
	**BCF**	**7.555 ± 0.082**	—	—
C. albicans	SW	7.204 ± 0.100	49.63	0.4494
	OW	7.494 ± 0.092	1.73	1.0000
	LW	3.315 ± 0.064	>99.99	**<0.0001**
	CaW	4.298 ± 0.142	99.94	**<0.0001**
	CW	5.392 ± 0.030	99.22	**<0.0001**
	CrW	5.271 ± 0.144	99.41	**<0.0001**

RW	4.303 ± 0.063	99.94	**<0.0001**
JW	7.138 ± 0.042	56.73	0.1676
RCW	2.328 ± 0.105	>99.99	**<0.0001**
SOW	7.319 ± 0.068	34.44	0.9544
BBW	4.021 ± 0.070	99.97	**<0.0001**
PBW	3.934 ± 0.057	99.97	**<0.0001**
**BCF**	**7.502 ± 0.071**	**—**	**—**

Values are presented as mean ± standard error of the mean (SEM). Reduction (%) was calculated from the difference between the mean log_{10} CFU values of each treatment and the corresponding uncoated bacterial cellulose control (BCF). The values of $p < 0.05$ were considered statistically significant. Reductions exceeding 99.99% are reported as >99.99%. Wax formulation abbreviations are defined in Table 1.

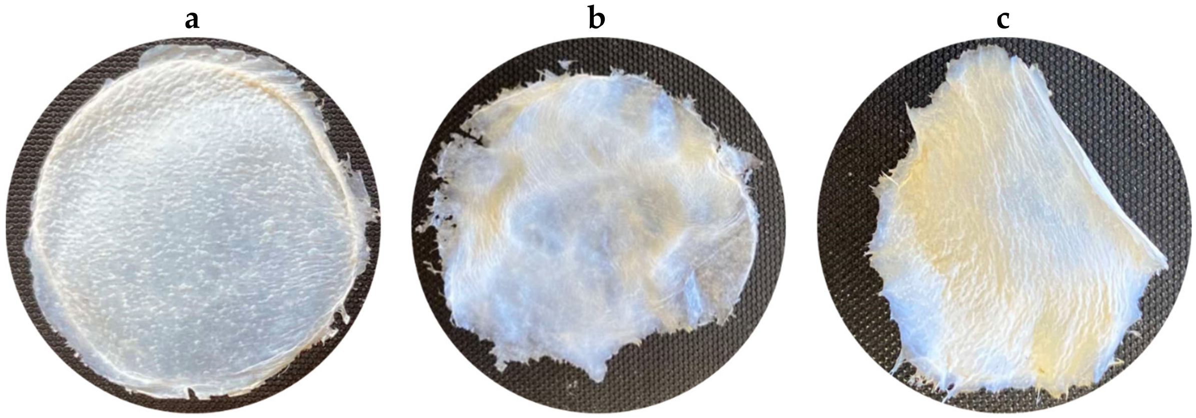

Figure S1. Macroscopic appearance of bacterial cellulose films (BCF) prepared at different drying temperatures: (a) 60 °C, (b) 70 °C, and (c) 80 °C. Images were acquired using a stereomicroscope.

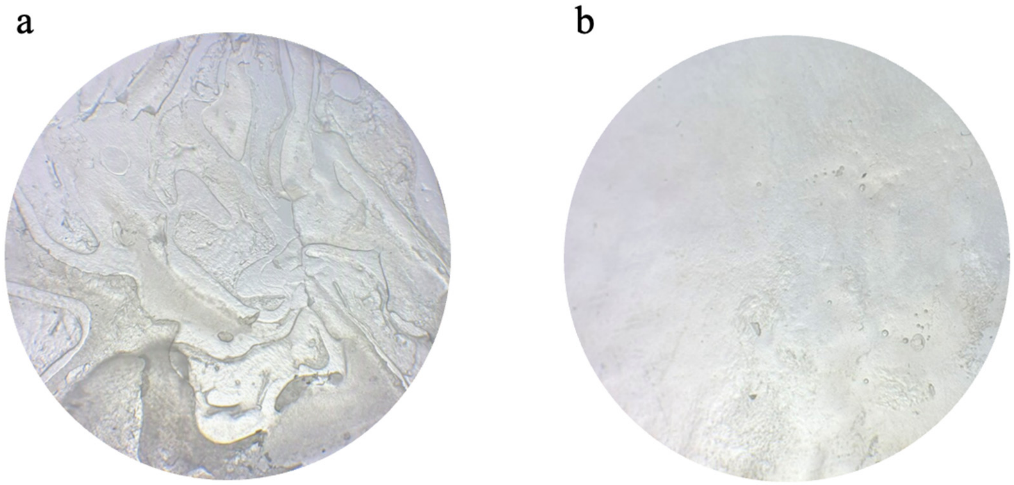

Figure S2. Representative differences in the surface spreading of wax formulations with different melting temperature ranges on bacterial cellulose films (BCF): (a) coconut wax (CW; 31–35 °C) and (b) rapeseed wax (RW; 65–75 °C).

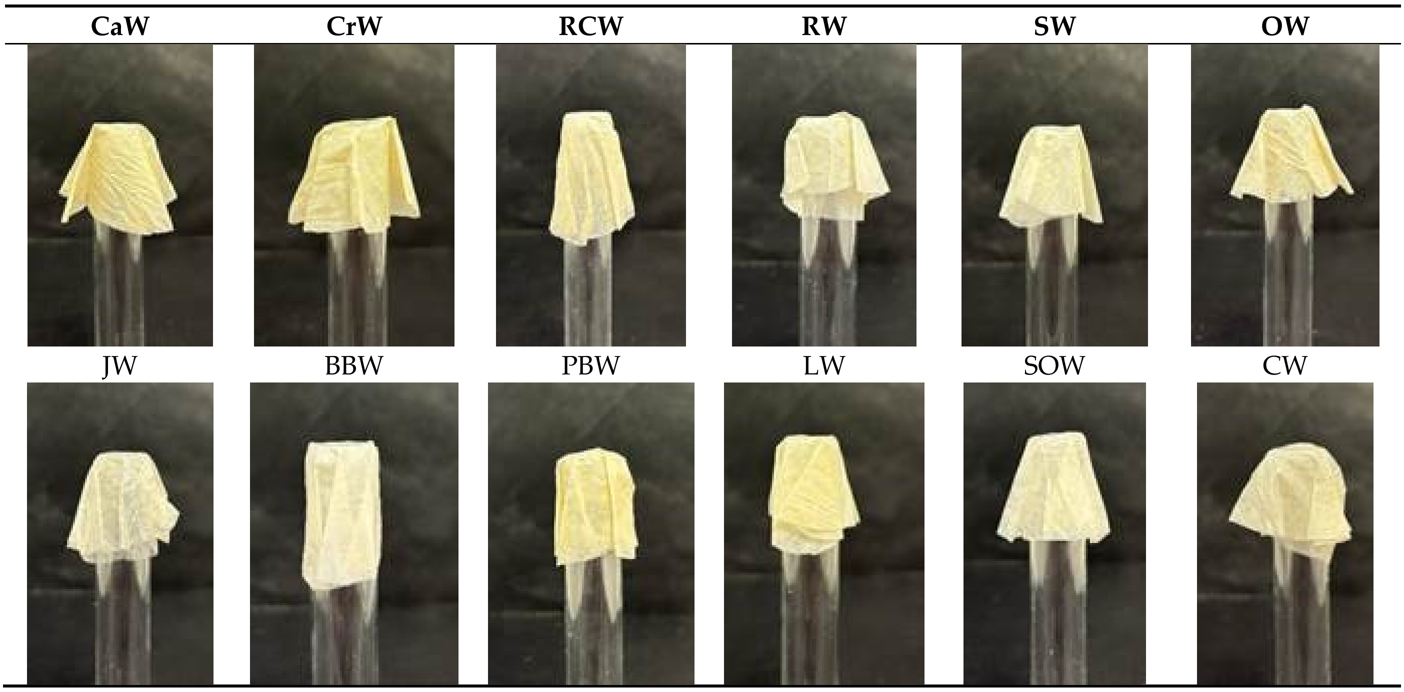

Figure S3. Qualitative assessment of the conformability of bacterial cellulose films coated with twelve commercial wax formulations (BCF-W) during manual application. Wax formulation abbreviations are defined in Table 1.
